# Protein profiling of fine‐needle aspirates reveals subtype‐associated immune signatures and involvement of chemokines in breast cancer

**DOI:** 10.1002/1878-0261.12410

**Published:** 2019-01-07

**Authors:** Bo Franzén, Andrey Alexeyenko, Masood Kamali‐Moghaddam, Thomas Hatschek, Lena Kanter, Torbjörn Ramqvist, Jonas Kierkegaard, Giuseppe Masucci, Gert Auer, Ulf Landegren, Rolf Lewensohn

**Affiliations:** ^1^ Department of Oncology and Pathology Cancer Center Karolinska Karolinska Institutet and University Hospital Stockholm Sweden; ^2^ Department of Microbiology, Tumor and Cell Biology (MTC) Karolinska Institutet Stockholm Sweden; ^3^ National Bioinformatics Infrastructure Sweden Science for Life Laboratory Solna Sweden; ^4^ Department of Immunology, Genetics and Pathology Science for Life Laboratory Uppsala University Sweden; ^5^ Theme Cancer Patient Area Head and Neck, Lung, and Skin Karolinska University Hospital Stockholm Sweden; ^6^ BröstCentrum City Stockholm Sweden; ^7^ Capio S:t Görans Sjukhus Stockholm Sweden

**Keywords:** breast cancer subtypes, fibroadenomas, fine‐needle aspiration, immune‐related protein biomarker, proximity extension assay

## Abstract

There are increasing demands for informative cancer biomarkers, accessible via minimally invasive procedures, both for initial diagnostics and for follow‐up of personalized cancer therapy, including immunotherapy. Fine‐needle aspiration (FNA) biopsy provides ready access to relevant tissue samples; however, the minute amounts of sample require sensitive multiplex molecular analysis to be of clinical biomarker utility. We have applied proximity extension assays (PEA) to analyze 167 proteins in FNA samples from patients with breast cancer (BC;* n* = 25) and benign lesions (*n* = 32). We demonstrate that the FNA BC samples could be divided into two main clusters, characterized by differences in expression levels of the estrogen receptor (ER) and the proliferation marker Ki67. This clustering corresponded to some extent to established BC subtypes. Our analysis also revealed several proteins whose expression levels differed between BC and benign lesions (e.g.*,* CA9, GZMB, IL‐6, VEGFA, CXCL11, PDL1, and PCD1), as well as several chemokines correlating with ER and Ki67 status (e.g., CCL4, CCL8, CCL20, CXCL8, CXCL9, and CXCL17). Finally, we also identified three signatures that could predict Ki67 status, ER status, and tumor grade, respectively, based on a small subset of proteins, which was dominated by chemokines. To our knowledge, expression profiles of CCL13 in benign lesions and BC have not previously been described but were shown herein to correlate with proliferation (*P *= 0.00095), suggesting a role in advanced BC. Given the broad functional range of the proteins analyzed, immune‐related proteins were overrepresented among the observed alterations. Our pilot study supports the emerging role of chemokines in BC progression. Due to the minimally traumatic sampling and clinically important molecular information for therapeutic decisions, this methodology is promising for future immunoscoring and monitoring of treatment efficacy in BC.

**Abbreviations**
BC: breast cancer
ER: estrogen receptor, ESR1
ERBB2: receptor tyrosine‐protein kinase erbB‐2
FNA: fine‐needle aspiration biopsy
HER: HER2
HER: HER2‐positive
IHC: immunohistochemistry
Ki67: antigen Ki‐67
LumA: BC subtypes luminal A
LumB: luminal B
LumHER: luminal HER2‐positive
MWL: maximal whisker length
NPX: normalized protein expression
PEA: proximity extension assay
PR: progesterone receptor
RIPA: radioimmunoprecipitation assay buffer
TNB: triple‐negative breast cancer

## 1 Introduction

One of the main current challenges for breast cancer (BC) therapy is to device methods that allow repeated and minimally invasive sampling with the capacity to identify informative molecular signatures. This may provide support for the choice of neoadjuvant and/or immune therapy, and a possibility to monitor the disease characteristics during the course of the disease. In addition to classical BC biomarkers, such as ER, PGR, HER2, and Ki67 (referred to as ‘key properties’), there is a need for biomarkers reflecting the microenvironment and immunodynamics in BC. Therefore, considerable efforts are made to implement measurement of biomarkers via liquid biopsies of peripheral blood. However, tumor sampling via fine‐needle aspiration (FNA) provides direct access to tumor tissue material. FNA sampling is a well‐established cancer diagnostic procedure, where cells, tissue fragments, and/or fluid may be recovered from tumor tissue via a puncture using a very thin needle. The tip of the needle is placed in the center of a lesion, frequently under ultrasound guidance (Ly *et al*., 2016; Rimsten *et al*., 1975). Cells can then be aspirated via a syringe during gentle oscillation back and forth. The minimally traumatic FNA is often used for diagnosis of small nonpalpable, deeply located, or otherwise hard‐to‐reach lesions, both for primary tumors and for lymph node and other metastases. Traditionally, these samples have been subjected to cytological analysis with staining for a small number of proteins. By contrast, modern molecular technologies allow more comprehensive analyses of the state of the sampled tissues.

In this pilot study, we have explored one FNA‐compatible technology for multiplex molecular profiling that meets stringent requirements for sensitivity and reproducibility: proximity extension assays (PEA, www.olink.com; Assarsson *et al*., 2014).

We have previously demonstrated that ‘leftover’ FNA sample material from BC can be analyzed by PEA with high sensitivity, and with results that correlated with routine assessments. In addition, our results revealed a tentative 11‐protein signature that discriminated BC from benign lesions (Franzén *et al*., 2018).

In this study, we have used the same patient material analyzed with the same protein panels as previously described, to further explore the protein expression in benign lesions in relation to BC subtypes and to several routine parameters such as proliferation and hormonal status. The two protein panels used in these studies—Oncology II and Immuno‐Oncology I (https://www.olink.com/products/immuno-oncology/)—include many proteins that may be relevant for characterization of clinical and biological properties of BC, including proteins involved in immune reactions. Twenty‐seven of the proteins of the ‘Oncology II’ panel and 66 proteins of the ‘Immuno‐Oncology I’ panel are immune‐related according to their annotations [https://www.olink.com/products/]. Due to some overlap between the two panels, the analysis measured levels of a total of 167 different proteins. We applied three complementary statistical approaches to analyze data relative to BC subtypes, and to histological/immunohistochemical (IHC), clinical, and cytology information: (a) hierarchical clustering of protein profiles to examine BC subtypes, (b) multiple‐regression modeling of signatures linking to key properties of BC, and (c) univariate correlations between protein levels and key properties of BC.

The long‐term aim of the study was to explore the potential value for FNA‐based molecular profiling by PEA for diagnosis, therapy selection, and evaluation in an objective and cost‐effective manner for BC patients. Our working hypothesis was that the analysis of key immune signatures in FNA samples using a simplified process may provide a valuable basis for choice of therapy and evaluation of responses to treatment.

## 2 Materials and methods

The materials and methods have been described previously (Franzén *et al*., 2018) and are briefly given below.

### 2.1 Patient samples

The study was approved by the Ethical Committee of the Karolinska Institutet, Stockholm (Dnr 2016/1432‐31/4), and the methodologies conform to the standards set by the Declaration of Helsinki. Female patients from the age of 18 years with mammography‐detectable lesions were invited to participate in the study after taking part of the project information and accepting the informed consent form. The patient cohort was subject to routine diagnostic examination (primary lesions in the breast and axillary metastases), and the material was collected during a period of 6 weeks. All FNA samples were obtained under ultrasound guidance by experienced radiologists using 21‐ to 22‐gauge needles, and after sampling for routine cytology, leftover material from FNA needles was processed immediately. For complete information on all samples, see Tables S1–S3.

### 2.2 Sample preparation

After sample collection, the FNA needles were rinsed with ice‐cold medium, and cells were pelleted, frozen, and stored at −80 °C until approval and quality control by cytology. Up to three samples were collected per patient, and each sample was assigned a consecutive FD# sample code. Samples approved by cytological examination were lysed in RIPA buffer (Sigma, Stockholm, Sweden R0278), debris was removed by centrifugation at 13 000 ***g*** for 15 min, and total protein concentration was determined using a micro‐BCA assay (ThermoFisher, Göteborg, Sweden).

### 2.3 Subtype classification

Routine core needle biopsy (CNB) tissue samples from primary lesions of BC patients, acquired in parallel with the FNA samples, were used for IHC analysis of estrogen receptor (ER), progesterone receptor (PR), the proliferation marker Ki67, and HER2 (ERBB2) according to routine guidelines. Classification of molecular subtypes was based on recommendations according to the St. Gallen classification system (Goldhirsch *et al*., 2011) and defined by the Swedish National Guidelines for treatment of BC (‘Nationella Vårdprogrammet för bröstcancer’, version 2.0, SweBCG 2018 [in Swedish; https://www.cancercentrum.se/samverkan/om-oss/nyheter/2018/februari/nationellt-vardprogram-brostcancer-uppdaterat/]) and the Quality and Standardization Committee (KVAST) of the Swedish Society of Pathology (2018, in Swedish).

### 2.4 Quality control and characterization by cytology

Routine cytology smears were stained (May–Grünwald–Giemsa), scanned at high resolution, and examined by an experienced cytopathologist. Each sample was checked for being representative and of sufficient quality by cytology evaluation. Samples without tumor cells or otherwise nonrepresentative were excluded. At least 20 different areas per preparation were examined for tumor cells and macrophages (MØ). Semiscore evaluation was performed as follows: If no MØ was found in a sample, the MØ score was set to = 0; if only one MØ was found in a sample, the MØ score was set to = 1; and if 2–3 MØ were found, the MØ score was set to = 2. If MØ were found in about 50% of fields examined, the MØ score was set to = 5, and if MØ were found in about > 80% of fields examined, the MØ score was set to = 10.

### 2.5 Protein profiling by PEA

One microlitre sample (0.5 μg total protein) per panel was analyzed by Olink Multiplex Immuno‐Oncology I and Oncology II panels (Olink Proteomics, Uppsala, Sweden) according to the manufacturer's instructions and as described previously (Larsson *et al*., 2015). Each panel consists of 92 protein assays and four internal controls. However, due to some overlap between the panels, in total data for 167 proteins were recorded (Table S6). Results were exported from the Biomark reader and normalized using Olink Wizard for GenEx software for further statistical data analysis.

### 2.6 Statistical analysis

Quality control and data preprocessing (including normalization) of PEA data were done in accordance with the manufacturer's recommended procedures, including the routine Olink normalization into NPX values, and the analysis was performed in R environment. We log_2_‐transformed the values to render them normally distributed (e.g., for Pearson linear correlation and ANOVA). Since clinical and other phenotypical variables were not necessarily normally distributed, we applied nonparametric statistics, such as chi‐square tests, Fisher's exact test, and Spearman's and Kendall's rank correlation, as indicated for the respective results. The *P*‐values from the correlation analyses were either adjusted by Benjamini–Hochberg correction (Benjamini and Hochberg, 1995), or by Bonferroni (Tables 2 and S5), or are shown as original unadjusted *P*‐values (Fig. 3B)—as specified in respective legends. The two‐way hierarchical clustering was performed on normally distributed protein profiles as well. The input to the PEA data analysis were the normalized protein expression values (NPX) as specified by Olink Proteomics. For the lasso/ridge multiple‐regression modeling, we employed package glmnet (available from http://web.stanford.edu/~hastie/glmnet/glmnet_alpha.html). In the latter analysis, parameter alpha equaled 1, while other parameters were set to their defaults. The two‐way hierarchical clustering was also performed on the normally distributed protein profiles. For this hierarchical clustering (shown at the heatmap figures), we employed the function heatmaply (R package heatmaply v. 0.15.12 https://cran.r-project.org/web/packages/heatmaply/) using the default methods for distance matrix calculation (‘euclidean’) and clustering (‘complete linkage’).

## 3 Results

### 3.1 Patient cohorts and PEA analysis

In total, 38 samples from 33 patients with benign breast lesions and 34 samples (≥ 8 mm in size) from 25 BC patients were analyzed for protein expression by PEA as described previously (Franzén *et al*., 2018). All information on patients and samples is listed in Tables S1–S3. For an overview of samples and IHC‐based subtype assignment, see Table 1.

**Table 1:** Overview of samples from benign lesions and cancers according to subtype. The molecular subtypes were defined according to the following criteria: ‘LumA’, luminal A‐like (ER‐ and/or PR‐positive, i.e., more than 10% positive cells; low Ki67, i.e., < 25% positive cells; and HER2‐negative); ‘LumB’, luminal B‐like HER2‐negative (ER‐positive and/or PR‐positive, and high Ki67, i.e., more than 25% positive cells; and HER2‐negative, i.e., 0 or 1+ according to IHC); ‘LumHER’, luminal B‐like HER2‐positive (ER‐positive and/or PR‐positive, any value for Ki67, and HER2‐positive, i.e., 2+ or 3+); ‘HER’, HER2‐positive, nonluminal (ER‐negative, PR‐negative, any value for Ki67, and HER2‐positive, confirmed by HER2 amplification using routine FISH technology when IHC is 2+ or 3+); and ‘TNB’, triple‐negative (ER‐ and PR‐negative, HER2‐negative, and any Ki67)

No. of patients	Sample types. Subtype based on IHC (St. Gallen)	Grade	No. of samples for PEA	No. of patients with two samples	No. of patients with three samples
Total: 32	Benign lesions	NA	38	6	
Total: 25	Cancer subtypes				
9^a^	Luminal A (LumA)	I–II	14	3^b^	1
4	Luminal B (LumB)	II–III	5	1	
5	Luminal HER (LumHER)	II–III	6	1^b^	
3	Nonluminal HER (HER)	III	5	2^b^	
4	TNB (TNBC)	II–III	4	0	
	Cancer samples		34	7	3

^a^Two of nine patients were diagnosed with lobular cancer. All other were ductal cancers. ^b^Includes one patient with samples from both primary tumor and axillary lymph node metastasis.

### 3.2 Hierarchical clustering of protein profiles

To explore similarities between samples from BC patients, protein profiles were compared by hierarchical clustering (Fig. S1). Pairwise correlations between sample profiles were calculated using PEA values for 134 proteins, that is, after excluding 33 proteins for which > 25% of the sample values were below the protein‐specific limits of detection (LOD; Table S6). The between‐samples clustering showed that the same or similar IHC subtypes clustered closer with each other. Also, when several multifocal (MF) lesions from the same patients were available, these clustered next to each other. There was one exception from the latter: One sample was diagnosed as ductal carcinoma *in situ* (DCIS, #FD93), while the other sample from the same patient was diagnosed as invasive ductal carcinoma (IDC). There were three cases where both primary tumor (P) and lymph node metastases (M) from the same patient were analyzed. In two out of these three, P and M failed to cluster adjacent to each other. All the three cases of lobular cancer were found in Cluster #2. Clusters #1a and #1b represent 53% ER‐negative samples, and Clusters #1c and #1d represent 100% ER‐positive samples. Thus, the protein‐based clustering indicated consistence with IHC subtypes. Interestingly, the sample #FD11 (Cluster #2) represented a small 12‐mm lesion, mammography code = 2 (i.e., presumably benign), but received triple‐negative breast cancer (TNB) as the final diagnosis. The remaining TNBs had mammography code = 5 (i.e., cancer) and being 25–40 mm in size; all ended up in Clusters #1a and #1b.

**Figure 1:**
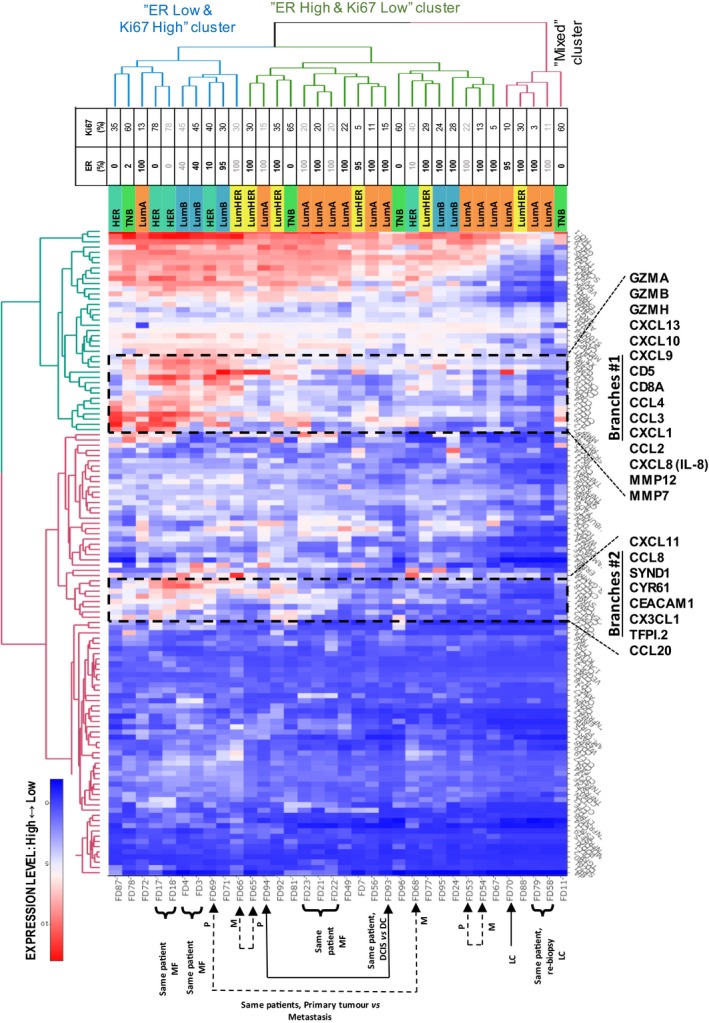
Heatmaps of protein expression for the patient samples: Two‐dimensional hierarchical clustering of samples vs protein profiles. Proteins for which > 25% of the values were below LOD were excluded. Compare Fig. S1 which shows a very similar clustering of samples. Clustering of proteins and samples indicates correlations between subtypes and protein functions. A zoom‐in view of Branches #1 is shown in Fig. S2. An interactive representation providing data values can be explored at http://research.scilifelab.se/andrej_alexeyenko/downloads/PEA/heatmap.PEA.34x124.v10.html.

To explore how proteins levels could determine sample clustering, we applied a two‐way hierarchical clustering of samples vs protein profiles (Fig. 1). Again, samples tended to cluster according to subtype. One cluster labeled ‘ER Low & Ki67 High’ had only 10% (1/10) samples of LumA type, while another ‘ER High & Ki67 Low’ cluster included 53% (10/19) samples of LumA type. A third cluster (‘Mixed cluster’) contained all three samples with lobular cancers. The first two clusters are significantly associated with different BC subtypes (chi‐square test, *P *< 0.05). ER and Ki67 expression values as determined by IHC (% positive cells) are significantly different between the first two clusters (*t*‐test, *P *< 0.05). In addition, the amount of MØ estimated by cytology was higher in the ‘ER Low & Ki67 High’ vs the ‘ER High & Ki67 Low’ cluster (*t*‐test, *P *< 0.05). In order to exclude possible influence of repeated samples available for a few patients in this analysis (i.e., multifocal cancers and metastases), we also performed clustering by leaving only one sample per patient. After reclustering, only two samples shifted cluster location compared to the previous clustering: #FD81 (TNB) and #FD92 (LumHER); both moved from the ‘ER High & Ki67 Low’ cluster to the ‘ER Low & Ki67 High’. Thus, clustering of samples seemed to be robustly correlated with biological features of subtypes and indicated the different biology between ductal and lobular cancers.

With respect to the protein dimension, we observed two prominent protein dendrogram branch groups (Branches #1 and Branches #2) that showed elevated expression levels in the ‘ER Low & Ki67 High’ sample cluster compared to the ‘ER High & Ki67 Low’ cluster. Notably, both Branches #1 and #2 were dominated by immune‐related proteins including in total twelve chemokines and three granzymes.

Dendrogram Branches #1 included the MØ/M2‐related protein MMP12 and several protumorigenic chemokines (e.g., CCL2, IL‐8/CXCL8, CXCL1), the T‐cell‐related markers CD8A and CD5, the angiogenic chemokines CXCL9 and CXCL10, and three granzymes known to be produced by cytotoxic T and NK cells (Lyons *et al*., 2017). A clustering pattern of samples was found, similar to that in Fig. S1. A zoom‐in view of the chemokine rich group of Branches #1 is shown in Fig. S2.

An estimation of the number of tumor‐infiltrating MØ showed that 80% (8/10) of samples in the ‘ER Low/Ki67 High’ cluster contained MØ (average score = 3.1). In contrast, only 31% (5/16) of samples in the ‘ER High/Ki67 Low’ cluster contained MØ (average score = 0.7). The MØ scoring revealed a significant difference between the two clusters (*P* = 0.011). Thus, high levels of MØ were more associated with the ‘ER Low & Ki67 High’ PEA phenotype and with TNB. All (4/4) TNB and 80% (4/5) HER samples contained MØ, while only 18% (2/11) in the LumA samples did. Cytology image samples from two patients with multifocal HER2 (FD17 and FD18) and multifocal LumA (FD21 and FD22) cancers are shown in Fig. S3. These representative subsets showed few or no MØ in the LumA samples, but greater numbers in the HER2 samples (MØ score = 5).

Given the key role of tumor‐infiltrating lymphocytes (TILs) in BC (Stanton and Disis, 2016), and the clustering neighbors to CD8A (as marker of CD8+ TILs), we performed a correlation analysis using CD8A protein values as a proxy reference of CD8+ TILs (Table S4). A correlation analysis of all protein profiles in all samples using CD8A as a reference confirmed significant correlation between several proteins (CD5, GZMBA, GZMH, CXCL10, CXCL9, GZMB, and CCL4) within Branches #1, increasing the support for higher CD8+ T‐cell‐related activity in samples representing the ‘ER Low & Ki67 High’ cluster (Table S4). The elevated granzyme levels also indicate the presence of activated CD8+ T cells.

The clustering of samples in Fig. 1 indicated clear differences in protein levels between the ‘ER High & Ki67 Low’ vs ‘ER Low & Ki67 High’ clusters. To explore differences specifically related to BC subtypes, the protein levels of ER‐negative (HER and TNB) cancers were compared to those representing the least aggressive IHC subtype, that is, LumA for the complete protein dataset (167 proteins).

There were significant differences (*t*‐test, *P* < 0.05) in 53 proteins, with the levels of 50 of these proteins increased in ER‐negative compared to LumA samples. Notably, CA9, IL‐8, CXCL17, CCL20, CCL7, CCl4, and IL‐6 all differed significantly upon Bonferroni correction, and among the top proteins in the fold‐change ranking list, there was an overrepresentation of cytokines (16 of top 34, odds ratio = 9.24, *P *= 0.000036, Fisher's exact test) demonstrating pronounced immune‐related differences between these subtypes. Twenty out of the top 32 proteins were included within the dendrogram Branches #1 or #2 (Fig. 1). Interestingly, the hypoxia marker CA9 and the MØ‐related protein MMP12 showed the highest fold increases in ER‐negative vs LumA samples (> 36‐fold), followed by the chemokines CXCL8, CCL2, CCL7, CXCL10, and CXCL9. All the latter proteins were > 10‐fold higher in ER‐negative vs LumA samples (Table S5).

### 3.3 Prediction of Ki67, ER status, and tumor grade by multiple‐regression protein level signatures

The next step in the analysis addressed whether protein profiles could be identified that correlated with key biological properties of BC, as this might reveal important proteins within the tumor microenvironment. Current subtyping of BC depends on subjective IHC‐based microscopic examination by experienced pathologists and, according to the standard protocols, involves *binary cutoffs* for ER, Ki67, and HER2 levels. Therefore, we explored an alternative approach to determine ER, Ki67, and HER2 levels along a continuous scale via protein signatures, potentially relevant to key biological properties of BC.

**Figure 2:**
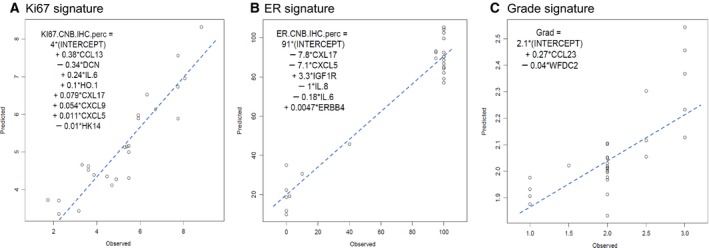
Regression models predictive for IHC‐based observed expression of Ki67 (A), ER (B), and tumor grade (C). Expression levels of chemokines CCL13, CXCL17, CXCL9, CXCL5, CXCL8 (IL‐8), and CCL23 contribute together with other proteins to key properties of BC. ‘Observed’ denotes the IHC values (Ki67 or ER IHC staining positivity) for each of the samples on a continuous scale (0–10, 0–100, or 0–3 for Ki67, ER, and tumor grade, respectively, *X*‐axis). ‘Predicted’ is the quantitative score assigned by the algorithm along the same continuous range (*Y*‐axis). Member proteins in the signature are described in the text.

We succeeded in producing significant predictive models for the Ki67 and ER IHC clinical variables and for the tumor grade (i.e., malignancy grade according to Nottingham histological grading), but not for the HER2 status (Fig. 2A–C). In these models, the predictive score for a given sample was calculated as a linear sum of protein expression values multiplied by the coefficients for each protein indicated in the plot legends. Surprisingly, these results also revealed that chemokines and/or cytokines strongly contributed to the modeling of all the three (ER, Ki67, and HER2) clinical variables.

The sets of significant proteins in the prediction models overlapped partially. Notably, the chemokines CXCL17 and CXCL5 and the cytokine IL‐6 contributed significantly to the prediction of both ER and Ki67 status, although with opposite signs of the linear coefficients (Fig. 2A,B). Specifically, CXCL17 and CXCL5 were increased in cancers with high proliferation as assessed by Ki67 and decreased in cancers with high ER levels. The chemokine CCL13 and decorin (DCN) provided strong contributions to the prediction of Ki67 status as previously described (Franzén *et al*., 2018). IGF1R, known to correlate with ER and good prognosis, contributed positively to the prediction of ER status. Proinflammatory cytokines IL‐6 and IL‐8 showed negative correlation with ER status, while IL‐6 correlated positively with Ki67 status, which indicated increased inflammation in ‘ER Low & Ki67 High’ cancers (Williams *et al*., 2016).

The regression model for the observed tumor grade used a combination of only two proteins, CCL23 and WFDC2 (Fig. 2C). The chemokine CCL23, also known as MØ inflammatory protein 3 (MIP‐3), is known as highly chemotactic for T cells and monocytes (Zlotnik and Yoshie, 2012). WFDC2 (WAP core domain protein 2), also known as human epididymis protein (HE4), has been proposed as diagnostic biomarker of ovarian cancer (Montagnana *et al*., 2009, p. 4).

Since the modeling was done on standardized protein values (i.e., expressed on the same scale), the magnitude of coefficients of linear multiple regression was informative regarding the relative contribution of the proteins in the regression models (i.e., the importance in the model). Results show that the top 3 proteins (CCL13, DCN, and IL‐6) contributed > 75% of the predictive value for proliferation, whereas three other proteins (CXCL17, CXCL5, and IGF1R) contributed > 75% of the information for the model predictive of ER status.

**Figure 3:**
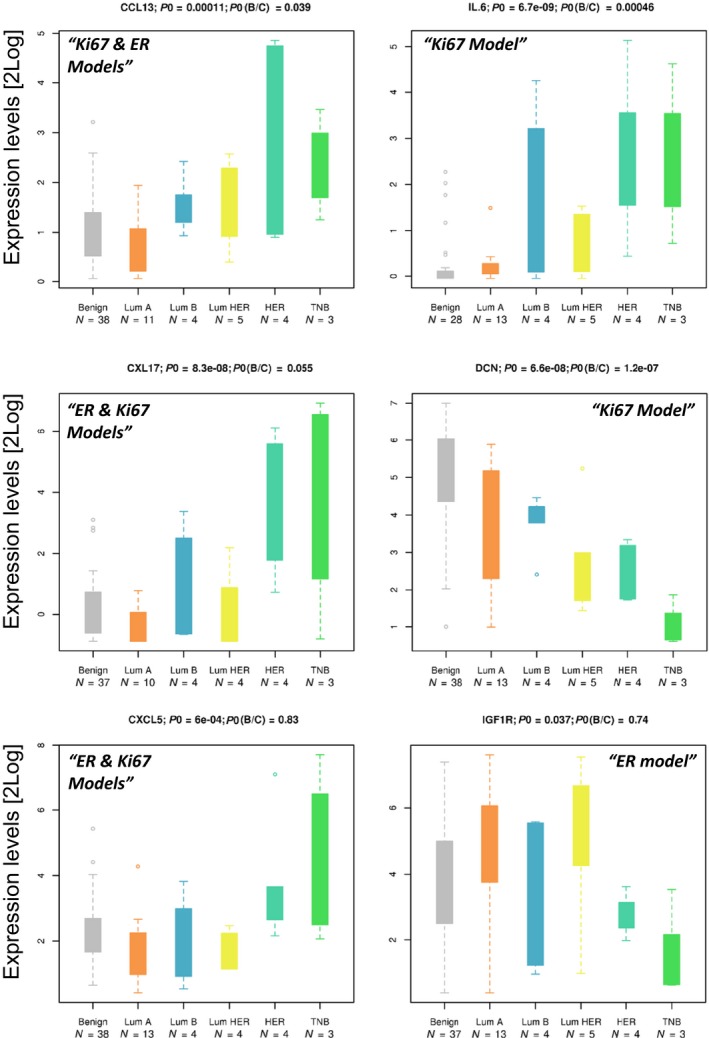
Expression levels of the six most prominent signature protein members in BC subtypes relative to baseline. Histograms show protein levels in benign lesions and cancer subtypes. The significance of differences is indicated by p0 for subtypes and p(B/C) for benign vs cancer. Individual samples are indicated by the FD sample numbers. Protein levels in different sample groups are also presented as boxplots. The protein levels expressed as NPX values are shown on a log_2_ scale where the boxes contain data points within 25–75th percentile intervals (i.e., between quartiles Q1 and Q3). The maximal whisker length (MWL) is defined as 1.5 times the Q1–Q3 interquartile range (i.e., the box length). Whiskers can extend either to the MWL or to the maximal available data point when the latter is below MWL. Markers thus correspond to data points that extend off the box by more than the MWL value. Sample numbers (FD#) are shown to indicate cases that repeatedly deviate from the main group, for example, the very early TNB case FD11 or the MØ enriched HER2 sample FD17.

Next, we examined the expression levels for the six most prominent protein members in the signatures for the BC subtypes, as well as in the benign lesions representing the baseline. The analysis demonstrated that expression of four of six proteins (CCL13, IL‐6, CXCL17, and DCN) also differed between the benign lesions and cancer (Fig. 3). Despite the obvious need for verification in larger cohorts, this result highlighted the potential roles of the six proteins in BC progression and the potential value of monitoring the balance between key proteins.

### 3.4 Correlations between immune‐related protein levels and BC key properties

To further explore correlations between protein levels and key properties of BC with respect to a continuous scale of IHC‐measured levels of Ki67, HER2, and ER, we applied a third approach to analyze *univariate* correlations for each of the 167 PEA assessed proteins to Ki67, HER2, and ER, and the tumor grade. Table 2, using rank correlations, displays proteins that significantly correlated with the aforementioned key properties, and for reference, proteins that also show significantly different levels between cancer and benign lesions (ANOVA). The far‐right column of Table 2 identifies leukocyte subsets associated with each protein, and other relevant information (for references, see Table 2 footnotes). We observed that the levels of 19/20 chemokines in the dataset were altered, a pronounced overrepresentation given that only 12% of all proteins analyzed belonged to this group of proteins (odds ratio = 13.3, *P *= 0.00097, Fisher's exact test). Interestingly, nine chemokines (CCL2, CCL3, CCL4, CCL8, CCL19, CXCL9, CXCL10, CXCL11, and CXCL13, marked by #) of 19 shown in Table 2 contribute to the 12‐chemokine signature described previously in BC and melanoma progression (Messina *et al*., 2012; Prabhakaran *et al*., 2017). Our results suggest that additional chemokines may be involved in the progression of BC, for instance, CCL13, CCL20, and CXCL17. Furthermore, several CD cell surface proteins correlated significantly with many of the key properties analyzed. For instance, the levels of CD8A (cytotoxic T‐cell marker) significantly correlated with tumor grade, Ki67 status, and ER status (*P *= 0.027, *P *= 0.00019, and *P *= 0.0022, respectively). Granzyme B (GZMB), known to be produced by CD8 T cells, showed significant correlation with the same variables. Moreover, several proteins associated with the M2 subset of MØ are positively correlated with the Ki67 status (e.g., CCL8, CCL13, CD4, MMP12, and ANG2).

**Table 2:** Rank correlations and increased (+) or decreased (−) protein levels in given BC subsets. B, B cells; DA, dendritic cells activated; DR, dendritic cells resting; MA, Mast cell activated; MØ, Macrophages; NKA, NK cells activated; NKR, NK cells resting; T4, CD4 naive; T4A, CD4 memory activated; T4R, CD4 memory resting; T8, T cells CD8; Tfh, follicular helper; Tgd, gamma delta; Th, T helper; Treg, regulatory

Chemokines	Cancer vs benign (1‐way ANOVA)	Grade (Kendall tau)	Ki67 (Spearman rank)	HER2 (Kendall tau)	ER (Spearman rank)	Comments and leukocyte subsets^a^
CCL2^b^	+ (*P* = 0.031)	+ (*P* = 0.017)	+ (*P* = 0.006)		− (*P* = 0.048)	Cluster. Th2/17/22^c^
CCL3^b^	+ (*P* = 0.0082)	+ (*P* = 0.013)	+ (*P* = 0.0099)		− (*P* = 0.025)	Cluster. Th1^c^
CCL4^b^	+ (*P* = 0.018)	+ (*P* = 0.016)	**+ (** ***P*** ** = 4.0e‐4)** *		− (*P* = 0.0048)	Cluster. NKA, MA, Th1/2/17/22^c^
CCL7	**+ (** ***P*** ** = 0.037)** *	+ (*P* = 0.0049)	+ (*P* = 0.037)			M0, Th1, Th2
CCL8^b^	**+ (** ***P*** ** = 0.037)** *	+ (*P* = 0.015)	**+ (** ***P*** ** = 5.7e‐4)** *		− (*P* = 0.021)	Cluster. M1, M2, DA, Th2
CCL13	+ (*P* = 0.039)		**+ (** ***P*** ** = 9.5e‐4)** *	+ (*P* = 0.016)		In model. M2, DR, DA, Th2
CCL17			+ (*P* = 0.036)			DR, DA, Th2/17/22
CCL19^b^	+ (*P* = 0.0011)					M1, DA
CCL20	+ (*P* = 0.0069)		**+ (** ***P*** ** = 7.4e‐5)** *		− (*P* = 0.0023)	Cluster. T4A, DA, MA, Th17/22
CCL23		+ (*P* = 0.0048)				In model. M2, Th1
CXCL1		+ (*P* = 0.021)	+ (*P* = 0.0086)		− (*P* = 0.042)	Cluster
CXCL5					− (*P* = 0.002)†	In model. M0
CXCL8 (IL‐8)	+ (*P* = 0.0077)		+ (*P* = 0.0063)		− (*P* = 0.017)	Cluster. Model. Inflammatory cytokine
CXCL9^b^	**+ (** ***P*** ** = 0.015)** *	+ (*P* = 0.018)	**+ (** ***P*** ** = 5.7e‐6)** *	+ (*P* = 0.010)	− (*P* = 0.0014)	Cluster. In model, M1
CXCL10^b^	**+ (** ***P*** ** = 0.0068)** *	+ (*P* = 0.039)	**+ (** ***P*** ** = 1.4e‐4)** *	+ (*P* = 0.018)	− (*P* = 0.013)	Cluster. M1, DA,
CXCL11^b^	**+ (** ***P*** ** = 0.0016)** *	+ (*P* = 0.033)	**+ (** ***P*** ** = 1.3e‐4)** *	+ (*P* = 0.029)	− (*P* = 0.012)	Cluster. M1, DA,
CXCL13^b^	− (*P* = 0.0018)		+ (*P* = 0.028)		− (*P* = 0.026)	Cluster. T4A, Tfh, M1^c^
CXCL17			**+ (** ***P*** ** = 2.2e‐5)** *		**− (** ***P*** ** = 2.8e‐4)** *	In model.
CX3CL1	− (*P* = 0.064)	+ (*P* = 0.020)	+ (*P* = 0.031)			Cluster
CD markers						
CD4		+ (*P* = 0.033)	+ (*P* = 0.027)			T4R, Treg, M2
CD5		+ (*P* = 0.017)	**+ (** ***P*** ** = 4.6e‐5)** *	+ (*P* = 0.0062)	− (*P* = 0.006)	Cluster. Treg
CD8A		+ (*P* = 0.016)	**+ (** ***P*** ** = 1.9e‐4)** *		− (*P* = 0.0022)	Cluster. T8, Tfh
CD27		+ (*P* = 0.017)	+ (*P* = 0.0033)			BC, T8, T4, T4R, Tfh, Treg
CD40		+ (*P* = 0.0033)	+ (*P* = 0.002)		− (*P* = 0.013)	M1
CD40L	+ (*P* = 0.016)		+ (*P* = 0.020)		− (*P* = 0.049)	T4, T4R, T4A, Tfh
CD48		+ (*P* = 0.0039)	**+ (** ***P*** ** = 3.1e‐4)** *	+ (*P* = 0.015)	− (*P* = 0.028)	
CD137 (TNFRSF9)			+ (*P* = 0.0055)		− (*P* = 0.013)	Treg
CD160	**− (** ***P*** ** = 0.024)** *					Tgd, NKR
CD208 (LAMP3)	+ (*P* = 0.0025)		+ (*P* = 0.017)		− (*P* = 0.036)	M1, DCA
CD244 (CD48L)		+ (*P* = 0.041)	+ (*P* = 0.0088)		− (*P* = 0.022)	Tgd, NKR, NKA, Treg
CD229 (LY9)		+ (*P* = 0.0079)	**+ (** ***P*** ** = 5.0e‐5)** *	+ (*P* = 0.031)	− (*P* = 0.019)	T8, T4, T4R, Tgd
CD258 (TNFSF14)		+ (*P* = 0.0037)	+ (*P* = 0.0017)	+ (*P* = 0.0431)	− (*P* = 0.025)	NKA, M0, apoptosis related
CD274 (PDL1)	+ (*P* = 0.0029)	+ (*P* = 0.016)				Th1, NK, T8, MØ
CD279 (PDCD1)	+ (*P* = 0.023)					Tfh
CD358 (TNFRSF21)	+ (*P* = 0.045)	+ (*P* = 0.031)	+ (*P* = 0.00044)		− (*P* = 0.0044)	Apoptosis related (DR6)
Other proteins						
IL‐6	+ (*P* = 6.4e‐4)		**+ (** ***P*** ** = 1.9e‐4)** *		**− (** ***P*** ** = 2.0e‐4)** *	In model. Inflammatory cytokine
IL‐18		+ (*P* = 0.024)			− (*P* = 0.017)	Inflammatory cytokine
GZMA		+ (*P* = 0.047)	**+ (** ***P*** ** = 7.3e‐5)** *		− (*P* = 0.0021)	Cluster. T8, T4R, Tgd, NKR, NKA
GZMB	+ (*P* = 0.018)	+ (*P* = 0.041)	**+ (** ***P*** ** = 1.4e‐5)** *		**− (** ***P*** ** = 1.7e‐4)** *	Cluster. T8, T4A, Tgd, NKR, NKA
GZMH		+ (*P* = 0.0059)	**+ (** ***P*** ** = 3.4e‐5)** *		− (*P* = 0.003)	Cluster. T8, Tgd, NKR, NKA
CA9	+ (*P* = 0.0061)		+ (*P* = 0.0021)		− (*P* = 0.0016)	Hypoxia related
FASL			+ (*P* = 0.0011)		− (*P* = 0.013)	Apoptosis related, NK
MMP12	+ (*P* = 0.0051)		+ (*P* = 0.0089)		− (*P* = 0.012)	Cluster. DCR, DCA, M2
VEGFA	**+ (** ***P*** ** = 0.036)** *	+ (*P* = 0.035)	+ (*P* = 0.0061)			Angiogenesis related, Treg
ANG2 (ANGPT2)	+ (*P* = 0.021)	+ (*P* = 0.042)	+ (*P* = 0.009)		− (*P* = 0.049)	Angiogenesis related, M1, M2
ESM1	+ (*P* = 0.0046)	+ (*P* = 0.045)	+ (*P* = 0.0015)		− (*P* = 0.026)	Angiogenesis related
PDGFB	+ (*P* = 0.0066)			+ (*P* = 0.040)		Platelets, Angiogenesis related
LYN		+ (*P* = 0.028)	**+ (** ***P*** ** = 3.8e‐4)** *		**− (** ***P*** ** = 2.3e‐4)** *	Proto‐oncogene, B
TCL1A		+ (*P* = 0.025)	+ (*P* = 0.024)			B, T8, NK (proteomicsdb.org)
GAL9 (LGALS9)		+ (*P* = 0.038)	+ (*P* = 0.004)		− (*P* = 0.015)	B, T4, NK (proteomicsdb.org)

^a^ *‘*Cluster’ (see Fig. 1B) or ‘Model’ (see Fig. 2A–C) refers to the analysis where the given protein was found to be altered. In addition, the following reports describe in which subset of leukocytes the respective proteins are expressed at elevated levels: Newman *et al*. (2015), Lyons *et al*. (2017), Strazza and Mor (2017), and Prat *et al*. (2017) and www.proteomicsdb.org. ^b^‘12‐chemokine gene signature’ includes CCL2, CCL3, CCL4, CCL5, CCL8, CCL18, CCL19, CCL21, CXCL9, CXCL10, CXCL11, and CXCL13 (Prabhakaran *et al*., 2017). ^c^Attract myeloid cells; see Turley *et al*. (2015). *P‐value in bold text: value after Bonferroni correction. ^†^ *P*‐value from the analysis of ER‐neg vs LumA samples (Table S5).

**Figure 4:**
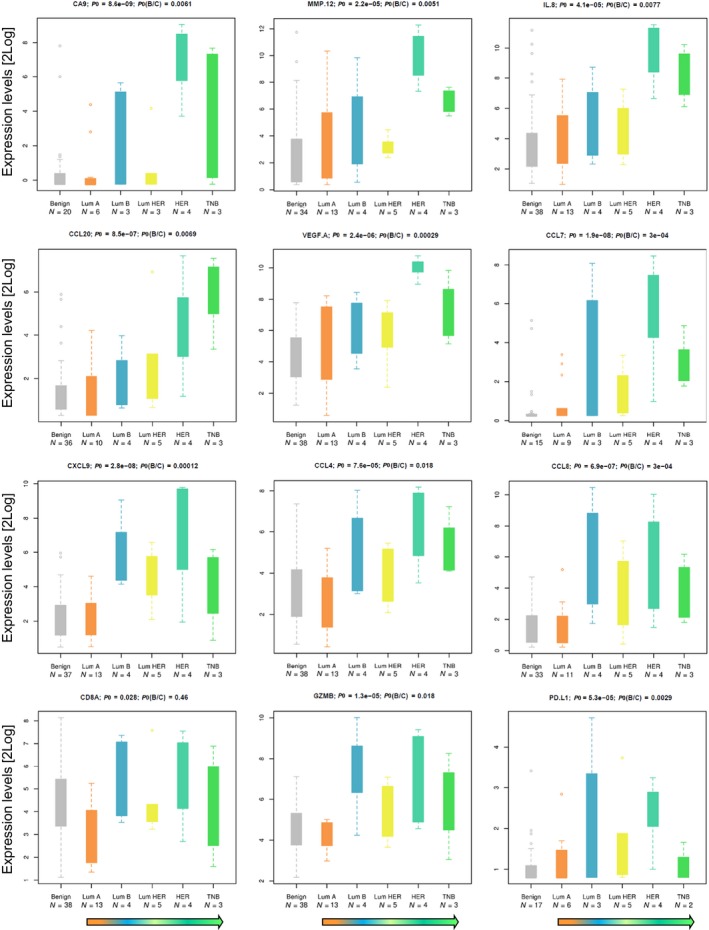
Protein correlations. A selection of proteins with correlation with grade or with the Ki67, HER2, or ER status, or with differences benign vs cancer, and/or LumA vs ER‐negative cancers (compare Fig. 3, Table 2, and Tables S4 and S5). Arrows at the bottom indicate expected decline of prognosis.

Complementary to Table 2, the histograms in Fig. 4 illustrate expression levels for a selection of 12 proteins from Table 2. The protein expression levels in BC subtypes are compared to those of benign lesions. All proteins except CD8A showed clear trends of increasing from benign (left bar) to more aggressive subtypes (HER2 and TNB, right bars) as indicated by the arrow at the far bottom of the boxplots. CD8A did not correlate clearly with BC subtype, although in LumA we observed lower levels of CD8A compared to other subtypes and benign lesions.

## 4 Discussion

We have previously reported that analysis using proximity extension assays on minute amounts of FNA samples in BC provides data that correlate with routine assessments of key markers such as ER, PGR, HER2, and Ki67. That study demonstrates that one microliter of lysates from FNA samples can be used for extensive targeted protein profiling. We used data from two PEA panels, ‘Oncology II’ and ‘Immuno‐Oncology I’, with a total of 167 unique proteins representing many different biological processes such as immunological mechanisms, cell adhesion, cell differentiation, cell motility, cell proliferation, apoptosis, and metabolic activity. In total, at least 80 of the proteins are directly immune‐related. Results showed that several immune‐related proteins, previously reported as important in therapy and progression of BC, can be quantified in a single FNA sample from minute material of primary lesions as small as 8 mm in size. In addition, several additional immune‐related proteins were found, of which many may be candidates for immune characterization of BC.

In the current study, we have evaluated expression levels of proteins in relation to routine parameters, that is, key properties and subtypes using various statistical approaches. Initially, hierarchical clustering of all protein profiles revealed two sample clusters, ‘ER High & Ki67 Low’ and ‘ER Low & Ki67 High’, with differences in protein levels, that is, proteins that may be related to expected aggressiveness of the tumors (i.e., ‘ER Low & Ki67 High’ cancers are expected to be more aggressive). We note that the difference between the two clusters showed an overrepresentation of immune‐related proteins, including many members from the chemokine family. The two dendrogram branch protein groups #1 and #2 involved 12 of a total of 20 investigated chemokines (60%), corresponding to only 12% of the total number of proteins analyzed. We therefore compared the more contrasting groups with respect to expected aggressiveness of the sampled tumors, namely LumA vs ER‐negative cases. The overrepresentation of chemokines in the protein profiles was confirmed, and in total, 16 chemokines showed significantly higher levels by on average 9.5‐fold in samples from ER‐negative vs LumA tumors. In this analysis, 13 of the top 21 proteins representing the highest fold changes were chemokines, and seven of these were part of the previously reported 12‐chemokine signature in malignant melanomas and BC (Prabhakaran *et al*., 2017). Our results indicate major differences in the involvement of different entities of the immune response between these subtypes. Given the correlation with clinical outcome, our results may be used to define a signature for future therapy selection in BC.

Interestingly, the MØ score was significantly higher in the ‘ER Low & Ki67 High’ cluster compared to the ‘ER High & Ki67 Low’ sample cluster (see Section 3.2). This difference coincides with the difference in chemokine levels between these two clusters (see Table S5). We observed a 36‐fold higher level of the protein CA9, a well‐established marker for hypoxia, and this may well be linked to several of the altered chemokine levels (Fig. 4). A reasonable hypothesis is that this overexpression is a consequence of recruitment of hypoxia‐driven tumor‐associated MØ (TAMs) and myeloid‐derived suppressor cells (MDSCs) to the tumor microenvironment and that this illustrates the ability of these cell types to differentiate to protumorigenic TAM subtypes. This hypothesis is supported by the observation of a higher level of MMP12 (36‐fold) and > 8‐fold higher levels of CCL2, CCL3, CCL4, and CXCL8, chemokines known to attract myeloid cells that differentiate into TAMs under hypoxia (Van Overmeire *et al*., 2014). Together with IL‐6, with a fivefold increased expression level, CCL2 may contribute to recruitment and polarization to protumorigenic M2‐like TAM phenotypes. Other chemokines, for example, CCL7, CCL20, CXCL1, and CXCL17, demonstrate similar expression patterns and may also be important contributors to MØ tumor infiltration, MØ differentiation, and a more aggressive phenotype.

Notably, among the top 11 proteins expressed at higher levels in the ‘ER High & Ki67 Low’ sample cluster, seven proteins were also expressed at elevated levels in HPV‐positive tonsillar and base of tongue cancer vs normal adjacent tissues (CA9, CXCL10, MMP12, CCL20, CXCL11, CXCL9, and CXCL8; Ramqvist *et al*., 2018). This indicates striking similarities in immune activation between the diverse tumor types.

A next level of analysis is provided by multiple‐regression modeling using continuous data for Ki67, ER, and tumor grade, rather than discrete data (i.e., BC subtypes). Three significant models were obtained that revealed protein signatures comprising in total 13 proteins. Here, the predominance of chemokines in the signatures, representing Ki67, ER, and grade, further highlights the apparently central role of chemokines in BC. Signatures show that expression levels of proteins in FNA samples from more aggressive BC were characterized by relatively high levels of CCL13, CXCL17, CXCL5, CCL23, and IL‐6, along with low levels of DCN and IGF1R. Less aggressive BC showed the inverse quantitative relationships for the same proteins. Taken together, the multiple‐regression models may reflect the quantitative balance between proteins in relation to a gradient from less aggressive (high ER, low Ki67, and low grade) to more aggressive (low ER, high Ki67, and high grade) phenotypes. Note also that for example, ERBB2, CD8A, and CD5 are not part of the signatures above. It is possible that the role of immune modulatory proteins has previously been underestimated in BC.

Our results clearly demonstrate that key immune‐related protein markers can be assessed in FNA material, for instance, TIL markers such as CCL2, CX3CL1, CXCL9, CXCL10, GZMA, GZMB, and GZMH (Galon *et al*., 2013). We confirm here results from previous studies that the expression levels of these markers and thereby TILs tend to be elevated in ER‐negative cancers compared to LumA cancers (Agahozo *et al*., 2018). However, the classical TIL marker CD8A alone did not differ between cancer and benign lesions and only weakly significant differences were observed between LumA and ER‐negative cancers (*P *= 0.018, non‐Bonferroni‐corrected *P*‐value).

Results presented herein also indicate that proteins that are components of the described signatures may play an important role in the biology behind the phenotypes, which traditionally is described in terms of ER, Ki67 expression, and tumor grade. In this context, it is surprising to note the prominent roles that chemokines seem to have in BC. The growth factor inhibitor DCN (decorin), which was previously shown to be decreased in BC compared to benign lesions (Franzén *et al*., 2018), interacts with IGF1R, EGFR, VEGFR2, ERBB2, and MMP7 (www.proteomicsdb.org), and studies also indicated that DCN may affect signaling via the chemokine receptor CXCR4 and is needed for autophagy.

The cytokine IL‐6 and chemokines CCL20, CXCL8, CXCL9, and CCL8 have previously been linked to overall survival in various cancers (Denkert *et al*., 2010; Farmaki *et al*., 2016, p. 8; Knüpfer and Preiß, 2007; Todorović‐Raković and Milovanović, 2013). However, several other chemokines have only to a limited extent been described previously in BC or benign breast lesions, for example, CXCL5, CXCL17, CCL13, CCL23, and CCL4. For instance, one recent report using PEA profiling of the extracellular compartment showed similar profiles in BC and mammographically dense healthy breast tissues compared to nondense healthy breast tissue; for instance, CCL4, CCL7, CCL8, CCL23, CXCL5, CXCL8, CXCL9, and VEGF showed elevated levels (Abrahamsson *et al*., 2018). Thus, the exact role of these cyto‐ and chemokines as assayed by PEA of FNA extracted tumor material for individual biological characteristics of BC needs further evaluation.

The chemokines CCL4 and CCL20 have been shown to recruit subsets of T cells in esophageal carcinoma, where CCL4 expression was correlated with the expression of CD8 and GZMB. This correlation was also observed in our study, which indicates a similar role in BC (Table S4; Liu *et al*., 2015). In contrast to CCL4, CCL20 has been shown to have several important functions in BC (Osuala and Sloane, 2014, p. 20).

Only one report so far describes a role for CXCL17 in BC. This is the most recent member of the chemokine family, and it is also known as VEGF coregulated chemokine 1. In this report, CXCL17 was found to be associated with shorter overall survival and thus represents a potential marker of poor prognosis (Guo *et al*., 2017).

Zhao *et al*. (2017) found that CXCL5 was overexpressed in tumor tissue and associated with poor prognosis in colorectal cancer patients. We are not aware of any previous reports on CXCL5 expression analysis in BC tissue. This also applies to CCL13 and CCL23 as the expression of these chemokines appears not to have been studied in BC tissue earlier, although CCL23 was found to be involved in angiogenesis (Hwang *et al*., 2005).

We have shown the correlation between immune markers such as CD8A and granzymes, CCL13, CCL20, CXCL17, CCL8, and CD4. These and others may represent immune contexture markers suggested as substitute to the classification and characterization of colon cancer by Galon *et al*. (2013). The finding illustrates the increasing potential to use the FNA to analyze predictive biomarkers in validation programs and clinical trials, as demonstrated recently (Foukakis *et al*., 2018).

Taken together, our data provide increased support for an association between chemokines and BC progression. The expression of cytokines in tumor tissues may be a manifestation of immune oncological host responses to the malignant cells, representing attempts to eradicate the tumor. On the other hand, chemokines, while less potent mitogens than growth factors, may also have a direct role in the proliferation of epithelial cancers. Oncogenes may activate chemokine receptor genes indirectly by regulating transcription factors involved in upregulation of proinflammatory chemokines. For instance, activation of chemokine receptors initiates intracellular signals leading to proliferation via ERK1/2 activation and the PI3K or β‐catenin pathways. Activation of chemokine receptors may also affect proliferation indirectly through transactivation of the epidermal growth factor receptor (EGFR) (Lacalle *et al*., 2017).

This is to our knowledge the first report on multiplex analysis of key immune‐related proteins in FNA samples from patients with primary BC. Our study demonstrates the feasibility to analyze immune‐related molecular signatures in minimal FNA samples as a possible avenue for future diagnostics, therapy selection, and monitoring of responses to therapy. This approach may also deliver new information about proteins with a role in BC progression and potential markers related to immunotherapy. Therefore, our results may be important for the future development of precision cancer medicine, for example, when considering neoadjuvant therapy or specific types of immunotherapy.

## 5 Conclusions

Immunological factors have proven significant predictors of neoadjuvant and adjuvant therapy responses in BC. It has previously been shown that several of these factors may be analyzed in FNA samples at the RNA level and that overexpression of immune‐related genes predicts chemosensitivity in patients with luminal advanced BC (Foukakis *et al*., 2018). However, the ultimate tumor phenotype is driven by the complex tumor microenvironment at the protein level, including products of genes expressed remotely from the sampled tissue and brought there via blood and thus undetectable at the level of mRNA in the tissue sample. Here, we show that a wide range of highly relevant immune‐related proteins can be analyzed in FNA samples, representing a snapshot of the native tumor microenvironment, using a simple sample preparation protocol and semi‐automated PEA technology. This is a pilot study which represents a highly translational approach and which paves the way for a new concept for comprehensive immune scoring and longitudinal monitoring of therapy responses. In conclusion, the approach demonstrated herein offers improved opportunities for individualized therapy selection and immune therapy evaluation.

## Conflicts of interest

UL is founder, shareholder, and board member of Olink Proteomics. No other authors declare any conflicts of interest.

## Author contributions

BF, MKM, TH, RL, UL, and GA were responsible for project design and infrastructure. JK was responsible for patient selection and sampling of material. LK and BF were responsible for scoring of macrophages by cytology. AA was responsible for data analysis, statistics, and bioinformatics. BF was responsible for writing the manuscript with support from all co‐authors.

## Supporting information


**Fig. S1.** Heatmap representing a two‐dimensional hierarchical clustering performed on a correlation matrix using the protein expression profiles of all cancer patient samples, that is, comparing all cancer samples against each other.
**Fig. S2.** Zoom‐in figure from Fig. 1b shows the chemokine rich cluster A.
**Fig. S3.** Example cytology samples from two patients with multifocal HER2 and multifocal luminal A cancers.
**Table S1.** Listing of all samples subjected to PEA and diagnosis by cytology (FNA material).
**Table S2.** Benign samples.
**Table S3.** Cancer samples from a total of 25 patients.
**Table S4.** Top 22 CD8A correlated proteins with reference to additional analysis results (see 3.3 and 3.4).
**Table S5.** PEA profiling of FNA samples reveals several significant (P<0.05) differences between samples from ER‐negative (HER2 + TNB) vs luminal A cases.
**Table S6.** Proteins in the PEA panels used (for more information: www.olink.com).
