# Supplementary material for: Protein profiling of fine‐needle aspirates reveals subtype‐associated immune signatures and involvement of chemokines in breast cancer
Source: Mol Oncol. 2019 Jan 7;13(2):376–91. doi: 10.1002/1878-0261.12410 (PMC6360506; doi:10.1002/1878-0261.12410)
Supplement: Supplementary file 1 — Fig. S1. Heatmap representing a two‐dimensional hierarchical clustering performed on a correlation matrix using the protein expression profiles of all cancer patient samples, that is, comparing all cancer samples against each other. Fig. S2. Zoom‐in figure from Fig. 1b shows the chemokine rich cluster A. Fig. S3. Example cytology samples from two patients with multifocal HER2 and multifocal luminal A cancers. Table S1. Listing of all samples subjected to PEA and diagnosis by cytology (FNA material). Table S2. Benign samples. Table S3. Cancer samples from a total of 25 patients. Table S4. Top 22 CD8A correlated proteins with reference to additional analysis results (see 3.3 and 3.4). Table S5. PEA profiling of FNA samples reveals several significant (P<0.05) differences between samples from ER‐negative (HER2 + TNB) vs luminal A cases. Table S6. Proteins in the PEA panels used (for more information: www.olink.com). [file MOL2-13-376-s001.docx]

**Supplementary Figures and Tables**

**Supplementary Figure 1. Heatmap representing a two-dimensional hierarchical clustering** **performed on a correlation matrix using the protein expression profiles of all cancer patient samples, *i.e.* comparing all cancer samples against each other.**

The color gradient represents the correlation between samples with respect to PEA profile where red equals high correlation and blue low correlation. Clusters show partial correlation to IHC subtypes and independent samples from multifocal (MF) cancer clusters adjacent to each other. For clarity, solid line boxes highlight the different clusters. An interactive representation, providing data values, can be explored at: <http://research.scilifelab.se/andrej_alexeyenko/downloads/PEA/pairwise_correlations.34samples.By_34_proteins.v3.html>


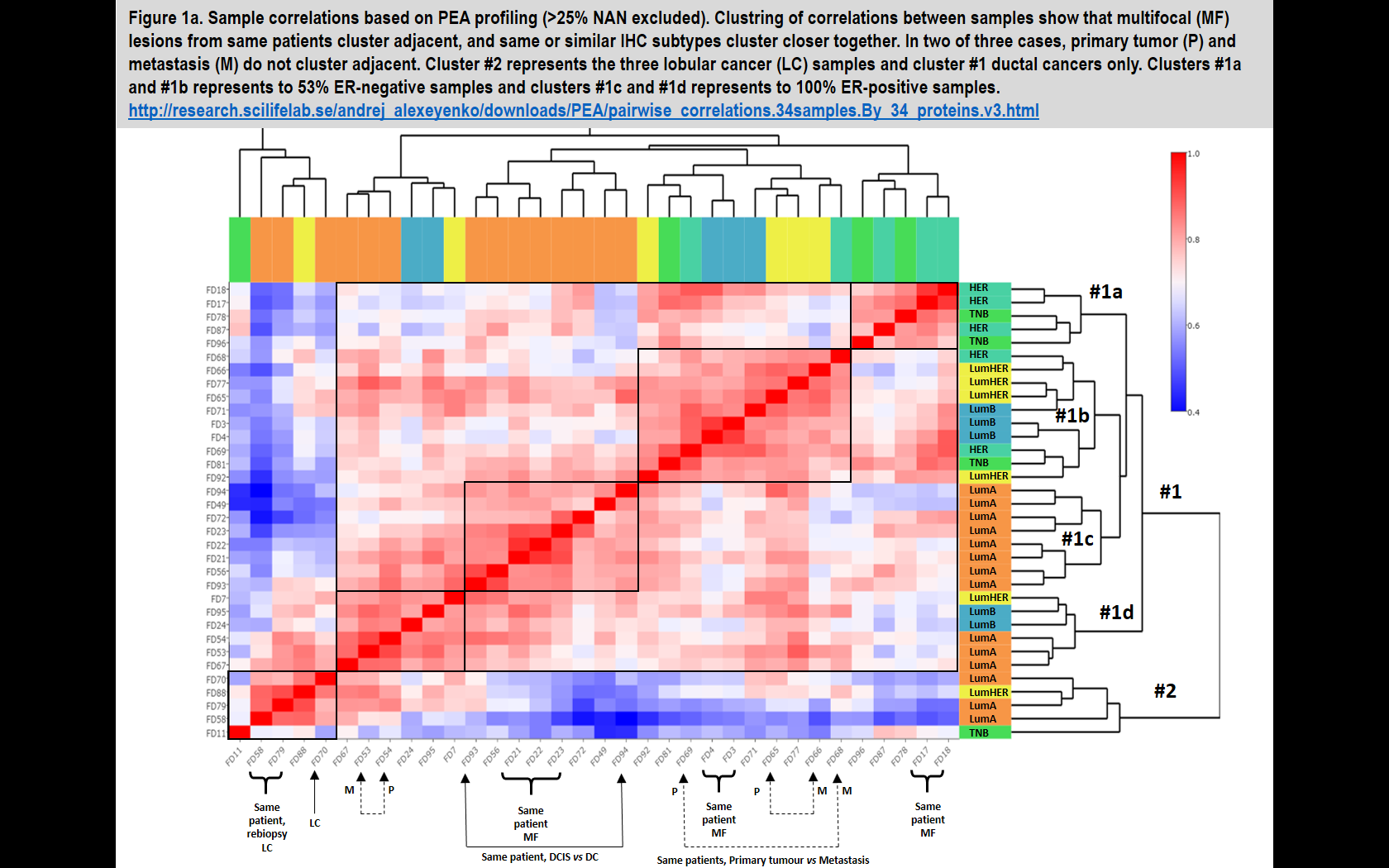


**Supplementary Figure 2. Zoom in figure from Figure 1b show the chemokine rich group of dendogram Branches #1.** Branches #1 include the macrophage-related protein MMP12 and pro-tumorigenic chemokines (*e.g.* CCL2, IL8/CXCL8), CXCL1), the T-cell related markers CD8A and CD5, the angiogenic chemokines CXCL9 and CXCL10, and three granzymes known to be produced by cytotoxic T-cells and NK-cells.

Sample clusters show correlation to IHC subtypes and samples from multifocal cancers (MF) cluster adjacent to each other (compare Figure 1a).

**Supplementary Figure 3. Examples of cytology samples from two patients with multifocal HER2 and multifocal Luminal A cancers.**

Routine cytology smears were stained (May-Grünwald Giemsa), scanned at high resolution and examined by an experienced cytopathologist. The upper row shows representative samples from patient (#109) with multifocal HER2 cancer (FD17 and FD18). The lower row shows representative samples from patient (#110) with multifocal Luminal A cancer (FD21 and FD22). At least 20 different areas per preparation were checked for tumor cells and macrophages (MØ, here indicated by arrow). Semiscore evaluation was performed as follows: If only one MØ was found in a sample, the MØ score was set to = 1, if 2-3 MØ were found, the MØ score was set to = 2, if MØ were found in about 50% of fields examined, the MØ score was set to = 5, and if MØ were found in in about > 80% of fields examined, the MØ score was set to = 10. The samples below show high number of MØ in the HER2 cancers (MØ score = 5) but MØ was almost completely absent in the Luminal A cancers (MØ score = 0-1). See also Materials & Methods.

**Supplementary Table 1.** Listing of all samples subjected to PEA and diagnosis by cytology (FNA material). Overview of all 58 patients, all samples and their final diagnoses (benign (n=33) and cancer (n=25) subtypes) according to IHC. Different shades of grey indicate multiple samples from the same patients (for details, see Tables 2 and 3). In three cases (#113, #115 and #117), axillary metastases (Ax. Met.) were sampled and all three cytology samples showed high concentrations of cancer cells. In two cases, benign and cancer samples were obtained from the same patient (pat. # 108 and #123). Text in *italics* *within square brackets* indicate samples where final diagnosis was obtained for a given patient was based on a parallel sample (multifocal tumors). In cases where multiple samples were obtained from a given patient, cytology and histology showed very high degrees of similarity. Therefore, we assign here the same cancer subtype to the parallel samples although the IHC analysis was performed on only one of the samples.

| **Patient  No.** | **Sample FD code** | **Final diagnosis** |
| --- | --- | --- |
| 101 | 12 | Benign |
| 101 | 13 | Benign |
| 102 | 24 | Lum B |
| 103 | 58 | *[Lum A, Lobular]* |
| 103 | 79 | Lum A, Lobular |
| 104 | 01 | Benign |
| 104 | 02 | Benign |
| 105 | 03 | *[Lum B]* |
| 105 | 04 | Lum B |
| 106 | 06 | Benign |
| 107 | 07 | Lum HER2 |
| 108 | 10 | Benign (FA) |
| 108 | 11 | TNBC |
| 109 | 17 | HER2 |
| 109 | 18 | *[HER2]* |
| 110 | 21 | Lum A |
| 110 | 22 | *[Lum A]* |
| 110 | 23 | *[Lum A]* |
| 112 | 49 | Lum A |
| 113 | 53 | *[Lum A] Ax. Met* |
| 113 | 54 | Lum A |
| 114 | 56 | Lum A (DCIS) |
| 115 | 65 | Lum HER2 |
| 115 | 66 | *[Lum HER2] Ax. Met* |
| 116 | 67 | Lum A |
| 117 | 68 | *[HER2] Ax. Met.* |
| 117 | 69 | HER2 |
| 118 | 70 | Lum A, lobular |
| 119 | 71 | Lum B |
| 120 | 72 | Lum A |
| 121 | 77 | Lum HER2 |
| 122 | 78 | TNBC |
| 123 | 81 | TNBC |
| 123 | 82 # | Normal lymph node (benign) |
| 124 | 87 | HER2 |
| 125 | 88 | Lum HER2 |
| 126 | 92 | Lum HER2 |
| **Patient  No.** | **Sample FD code** | **Final diagnosis** |
| 127 | 93 | Lum A |
| 127 | 94 | *[Lum A]* |
| 128 | 95 | Lum B |
| 129 | 96 | TNBC |
| 130 | 08 | Benign |
| 131 | 15 | Benign |
| 132 | 19 | Benign |
| 132 | 20 | Benign |
| 133 | 27 | Benign |
| 134 | 28 | Benign |
| 135 | 33 | Benign |
| 136 | 38 | Benign |
| 137 | 39 | Benign |
| 138 | 41 | Benign |
| 140 | 43 | Benign |
| 140 | 44 | Benign |
| 141 | 47 | Benign |
| 141 | 48 | Benign |
| 142 | 50 | Benign |
| 143 | 51 | Benign |
| 144 | 52 | Benign |
| 145 | 59 | Benign |
| 146 | 60 | Benign |
| 147 | 61 | Benign |
| 148 | 62 | Benign |
| 149 | 63 | Benign |
| 150 | 64 | Benign |
| 151 | 73 | Benign |
| 152 | 74 | Benign |
| 153 | 75 | Benign |
| 154 | 76 | Benign |
| 155 | 80 | Benign |
| 156 | 83 | Benign |
| 157 | 86 | Benign |
| 158 | 89 | Benign |
| 158 | 90 | Benign |
| *# Sample FD82 was excluded from data analysis* | | |

**Supplementary Table 2.** Benign samples. In total, 33 patients were included and analyzed by PEA. From each of 6 patients, 2 samples were obtained (multifocal lesions, grey shaded rows) and in 2 patients (#108 and #123, FD code marked by *) we obtained both benign and cancer samples (compare Tab. 4 and 5). N.d. equals “not determined”.

| **Sample FD code** | **Patient  No.** | **Patient Age** | **Lesion side** | **Mam. code** | **Final diagnosis** | **Other diagnostic information** | | |
| --- | --- | --- | --- | --- | --- | --- | --- | --- |
|  |  |  |  |  |  | **Fibro-adenoma** | **Fibro- adenosis** | **Hyperplasia** |
| 1 | 104 | 25 | Dex | n.d. | Benign | Yes |  |  |
| 2 | 104 | 25 | Sin | n.d. | Benign | Yes |  |  |
| 6 | 106 | 21 | Dex | 2 | Benign | Yes |  | Yes |
| 8 | 130 | 19 | Dex | 2 | Benign | Yes |  |  |
| 10* | 108 | 40 | Sin | 2 | Benign | Yes |  |  |
| 12 | 101 | 38 | Dex | 4 | Benign |  | Yes | Yes |
| 13 | 101 | 38 | Dex | 3 | Benign |  | Yes | Yes |
| 15 | 131 | 28 | Dex | 2 | Benign | Yes |  |  |
| 19 | 132 | 36 | Dex | 2 | Benign |  | Yes |  |
| 20 | 132 | 36 | Sin | 2 | Benign | Yes | Yes |  |
| 27 | 133 | 37 | Sin | 2 | Benign | Yes (CNB) |  | Yes (CNB) |
| 28 | 134 | 20 | Sin | 2 | Benign | Yes |  |  |
| 33 | 135 | 22 | Sin | 2 | Benign |  |  |  |
| 38 | 136 | 28 | Sin | 2 | Benign |  |  |  |
| 39 | 137 | 20 | Sin | 2 | Benign | Yes |  | Yes |
| 41 | 138 | 36 | Sin | 3 | Benign | Yes |  |  |
| 43 | 140 | 25 | Sin | 2 | Benign |  |  |  |
| 44 | 140 | 25 | Dex | 2 | Benign | Yes |  |  |
| 47 | 141 | 31 | Sin | 3 | Benign |  | Yes | Yes |
| 48 | 141 | 31 | Sin | 2 | Benign |  |  | Yes |
| 50 | 142 | 21 | Dex | n.d. | Benign | Yes |  |  |
| 51 | 143 | 32 | Sin | 2 | Benign | Yes (CNB) |  |  |
| 52 | 144 | 33 | Dex | 2 | Benign | Yes |  |  |
| 59 | 145 | 18 | Dex | 2 | Benign | Yes |  |  |
| 60 | 146 | 25 | Sin | 2 | Benign | Yes |  |  |
| 61 | 147 | 50 | Dex | 2 | Benign | Yes |  |  |
| 62 | 148 | 25 | Sin | 2 | Benign | Yes |  | Yes |
| 63 | 149 | 38 | Sin | 2 | Benign | Yes (CNB) |  |  |
| 64 | 150 | 32 | Dex | 2 | Benign | Yes |  |  |
| 73 | 151 | 31 | Sin | 2 | Benign | Yes |  |  |
| 74 | 152 | 33 | Dex | 2 | Benign | Yes |  |  |
| 75 | 153 | 52 | Sin | 5 | Benign (Papilloma) |  |  |  |
| 76 | 154 | 18 | Dex | 2 | Benign | Yes |  |  |
| 80 | 155 | 51 | Sin | 3 | Benign | Yes |  |  |
| 83 | 156 | 33 | Sin | 2 | Benign | Yes |  |  |
| 86 | 157 | 19 | Dex | 2 | Benign | Yes |  |  |
| 89 | 158 | 51 | Sin | 2 | Benign | Yes |  |  |
| 90 | 158 | 51 | Dex | 2 | Benign | Yes |  |  |

**Supplementary Table 3.** Cancer samples from a total of 25 patients. From 8 patients 2-3 samples were obtained per patient (marked by grey shaded rows): Four of these patients (#105, #109, #127 and #110) had multifocal lesions, 3 patients (#113, #115 and #117) had primary cancer and axillary metastases (rich in cancer cells according to cytology) and from one patient (#103) FNA sampling was repeated 11 days after the first sampling. From 2 patients (#108 and #123), benign and cancer samples were obtained. For most cases, final diagnosis and IHC was based on CNB samples obtained directly after FNA sampling. In cases marked by “¤” final diagnosis and IHC was based on post-surgery sampling. (Compare Tables 2, 3 and 4). Median tumor size was 20 mm, multifocal lesions and metastases included, and 20 samples were obtained from tumors <20 mm in diameter, nine samples from tumors 20-40 mm and five samples from tumors >40 mm (according to mammography and/or ultra-sound). The symbol @ represents axillary metastasis. The macrophage score (MØ) was given based on visual inspection of cytology specimens (materials and methods).

| **Patient  No.** | **Sample FD code** | **Patient Age** | **Lesion  side** | **Mam.  code** | **Tumor  size  [mm]** | **Final  diagnosis** | **Histo.  grade** | **Her2 status** | **FISH  Amp.** | **ER  (%)** | **KI67 (%)** | **PGR  (%)** | **IHC Subtype** | **Macrophage (MØ) Score** |
| --- | --- | --- | --- | --- | --- | --- | --- | --- | --- | --- | --- | --- | --- | --- |
| 105 | 03 | 36 | Dex | 5 | 45 | IDC | 3 | 1+ | N.D. | 40 | 45 | 0 | Lum B | 2 |
| 105 | 04 | 36 | Dex | 5 | 22 | IDC |  |  |  |  |  |  |  | 2 |
| 107 | 07 | 67 | Dex | 5 | 13 | IDC | N.D. | 2+ | No | 95 | 5 | 65 | Lum HER | na |
| 108 | 11 | 40 | Dex | 2 | 12 | IDC | 3 | 0 | N.D. | 0 | 60 | 5 | TNBC | 1 |
| 109 | 17 | 57 | Sin | 5 | 19 | IDC | 3 | 3+ | Yes | 0 | 78 | 0 | HER | 5 |
| 109 | 18 | 57 | Sin | 5 | 17 | IDC |  |  |  |  |  |  |  | 5 |
| 110 | 21 | 53 | Sin | 4 | 19 | IDC | 2 | 1+ | N.D. | 100 | 20 | 5 | Lum A | 0 |
| 110 | 22 | 53 | Sin | 4 | 14 | IDC |  |  |  |  |  |  |  | 1 |
| 110 | 23 | 53 | Sin | 4 | 17 | IDC |  |  |  |  |  |  |  | 0 |
| 102 | 24 | 67 | Sin | 5 | 20 | IDC | 2 | 1+ | N.D. | 100 | 28 | 100 | Lum B | 0 |
| 112 | 49 | 67 | Sin | 5 | 27 | IDC | 2 | 1+ | N.D. | 100 | 22 | 60 | Lum A | 0 |
| 113 | 53 | 83 | Dex @ | 5 | 10 | IDC |  |  |  |  |  |  |  | 0 |
| 113 | 54 | 83 | Dex | 5 | 18 | IDC | 2 | 0 | N.D. | 100 | 13 | 80 | Lum A | na |
| 114 | 56 | 77 | Dex | 4 | 13 | DCIS | 1 | 0 | N.D. | 100 | 11 | 100 | Lum A | na |
| 103 | 58 | 69 | Sin | 3 | 12 | ILC |  |  |  |  |  |  |  | na |
| 115 | 65 | 41 | Sin | 5 | 40 | IDC | 2 | 2+ | No | 100 | 30 | 85 | Lum HER | 0 |
| 115 | 66 | 41 | Sin @ | 5 | 14 | IDC |  |  |  |  |  |  |  | 1 |
| 116 | 67 | 76 | Dex | 5 | 32 | IDC | 2 | 0 | N.D. | 100 | 5 | 50 | Lum A | 0 |
| 117 | 68 | 46 | Sin @ | 5 | 30 | IDC |  |  |  |  |  |  |  | 1,5 |
| 117 | 69 | 46 | Sin | 5 | 85 | IDC | 3 | 3+ | Yes | 10 | 40 | 0 | HER | 0 |
| 118 | 70 | 48 | Sin | 4 | 60 | ILC | 2 | 0 | N.D. | 95 | 10 | 100 | Lum A | 3 |
| 119 | 71 | 48 | Sin | 5 | 45 | IDC | 2 | 0 | N.D. | 95 | 30 | 80 | Lum B | 3 |
| 120 | 72 | 43 | Sin | 5 | 15 | IDC | 1 | 0 | N.D. | 100 | 13 | 100 | Lum A | 0 |
| 121 | 77 | 86 | Dex | 5 | 20 | IDC | 2 | 2+ | Yes | 100 | 29 | 90 | Lum HER | 0 |
| 122 | 78 | 52 | Sin | 5 | 25 | IDC | 3 | 0 | N.D. | 2 | 60 | 0 | TNBC | 3 |
| 103 | 79 | 69 | Sin | 4 | 8 | ILC | 2 | 0 | N.D. | 100 | 3 | 0 | Lum A | 0 |
| 123 | 81 | 58 | Dex | 5 | 40 | IDC | 2 | 1+ | N.D. | 0 | 65 | 0 | TNBC | 2 |
| 124 | 87 | 71 | Dex | 5 | 70 | IDC/DCIS | 3 | 3+ | Yes | 0 | 35 | 0 | HER | 10 |
| 125 | 88 | 17 | Sin | 5 | 20 | IDC | 3 | 2+ | No | 100 | 30 | 0 | Lum HER | 0 |
| 126 | 92 | 71 | Sin | 5 | 20 | IDC | 3 | 2+ | No | 100 | 35 | 80 | Lum HER | 1 |
| 127 | 93 | 42 | Sin | 5 | 20 | IDC/DCIS | 1 | 0 | N.D. | 100 | 15 | 100 | Lum A | 0 |
| 127 | 94 | 42 | Sin | 5 | 10 | IDC |  |  |  |  |  |  |  | 2 |
| 128 | 95 | 89 | Dex | 5 | 38 | IDC | 2 | 1+ | N.D. | 100 | 24 | 95 | Lum B | 0 |
| 129 | 96 | 64 | Sin | 5 | 40 | IDC | 3 | 1+ | N.D. | 0 | 60 | 10 | TNBC | 5 |

**Supplementary Table 4.**

Top-22 CD8A correlated proteins with reference to additional analysis results (see 3.3 and 3.4)

The table show the results of protein profile correlation analysis of all cancer samples using CD8A as reference protein. This analysis confirms significant correlation between several proteins (*CD5, GZMBA, GZMH, CXCL10, CXCL9, GZMB and CCL4)* within dendogram branches A and indicate increased T-cell related activity in samples representing the “ER Low & Ki67 High” cluster. For reference we also include results from section 3.3. and 3.4

| **Protein** | **Correlation to CD8A levels** | **FDR (Corr.)** | **Part of  cluster** | **ER-corr.** (Univariate) | **Ki67-corr.** (Univariate) | **Grade-corr.** (Univariate) | **HER2** (Univariate) | **ER-negative vs LumA**  (Fold change) |
| --- | --- | --- | --- | --- | --- | --- | --- | --- |
| CD8A | 1.000 | 0 | A | (-) | + | (+) | ns | 3.40 |
| CD5 | 0.918 | 0 | A | (-) | + | (+) | (+) | 3.42 |
| GZMA | 0,893 | 7.69E-14 | A | (-) | + | (+) | ns | 2.68 |
| CD27 | 0.864 | 1.55E-11 |  | ns | (+) | (+) | ns | 1.98 |
| FASL | 0.849 | 1.32E-10 |  | (-) | (+) | ns | ns | 2.30 |
| TNFRSF9 | 0,844 | 2.61E-10 |  | (-) | (+) | (+) | ns | 3.19 |
| GZMH | 0.842 | 3.27E-10 | A | (-) | + | (+) | ns | 2.84 |
| CD48 | 0,836 | 6.61E-10 |  | (-) | + | (+) | ns | 1.74 |
| CXCL10 | 0.830 | 1.27E-09 | A | (-) | + | (+) | (+) | 10.8 |
| CXCL9 | 0,830 | 1.34E-09 | A | (-) | + | (+) | (+) | 10.4 |
| CD244 | 0.820 | 3.71E-09 |  | (-) | (+) | ns | ns | 1.92 |
| CD40 | 0,809 | 1.02E-08 |  | (-) | (+) | (+) | ns | 3.31 |
| LY9 | 0.803 | 1.81E-08 |  | (-) | + | (+) | (+) | 2.12 |
| GZMB | 0,802 | 1.88E-08 | A | - | + | (+) | ns | 5.64 |
| CCL4 | 0.791 | 4.89E-08 | A | (-) | + | (+) | ns | 8.62 |
| CD4 | 0,782 | 9.81E-08 |  | ns | (+) | ns | ns | - |
| PLGF | 0,781 | 1.05E-07 |  | ns | ns | ns | ns | - |
| NOS3 | 0,774 | 1.70E-07 |  | ns | (+) | ns | ns | - |
| CXCL11 | 0,760 | 4.66E-07 | B | (-) | + | (+) | (+) | 9.40 |
| CCL8 | 0.748 | 9.74E-07 | B | (-) | + | (+) | ns | 8.98 |
| LYN | 0,740 | 1.57E-06 |  | - | + | (+) | ns | 2.08 |
| TNFSF14 | 0.716 | 5.87E-06 |  | (-) | (+) | (+) | (+) | 2.33 |

(FDR= False Discovery Rate) (ns= not significant)

**Supplementary Table 5.**

PEA profiling of FNA samples reveal several significant (P<0,05) differences between samples from ER-negative (HER2 + TNB) vs Luminal A cases. Bonferroni correction was made to compensate for multiple testing, and the most significantly different proteins are highlighted in bold. We show the ranked fold-change list of all proteins without this correction of the P-value.

| **Protein (PEA)** | **Fold change PEA ER-neg *vs* Luminal A** | **T-test (p-value)** |
| --- | --- | --- |
| **CA9** | **36.83** | **5.60E-05 *** |
| MMP12 **^#^** | 36.38 | 0.00064 |
| **IL8 (CXCL8)** **^#^** | **24.83** | **3.40E-05 *** |
| **CXCL17 ^#^** | **15.58** | **2.60E-05 *** |
| **CCL20 ^#^** | **14.10** | **4.80E-05 *** |
| VEGFA | 12.70 | 0.0015 |
| CCL2 **^#^** | 12.00 | 0.0033 |
| **CCL7 ^U^** | **11.63** | **0.00031 *** |
| CXCL10 **^#^** | 10.84 | 0.013 |
| CXCL9 **^#^** | 10.37 | 0.0022 |
| CXCL11 **^#^** | 9.40 | 0.0023 |
| CCL8 **^#^** | 8.98 | 0.0025 |
| **CCL4 ^#^** | **8.62** | **0.00021 *** |
| CCL3 **^#^** | 8.43 | 0.0013 |
| MK (MDK) | 6.93 | 0.043 |
| SYND1 (SDC1) **^#^** | 6.63 | 0.0067 |
| CXCL1 **^#^** | 5.67 | 0.00071 |
| GZMB **^#^** | 5.64 | 0.00091 |
| **IL6** | **5.28** | **2.50E-05 *** |
| HO1 (HMOX1) | 5.11 | 0.024 |
| CXCL5 | 5.04 | 0.0018 |
| TCL1A | 4.63 | 0.015 |
| TFPI2 **^#^** | 4.40 | 0.029 |
| CEACAM1 **^#^** | 3.75 | 0.028 |
| CD5 **^#^** | 3.43 | 0.012 |
| CD8A **^#^** | 3.40 | 0.018 |
| CCL13 | 3.35 | 0.00086 |
| CD40 | 3.31 | 0.028 |
| TNFRSF9 | 3.19 | 0.011 |
| ESM1 | 2.93 | 0.0042 |
| GZMH **^#^** | 2.84 | 0.011 |
| GZMA **^#^** | 2.68 | 0.016 |
| CASP8 | 2.59 | 0.017 |
| IFN gamma R1 | 2.53 | 0.034 |
| PDGFB | 2.43 | 0.049 |
| TNFSF14 | 2.33 | 0.00076 |
| FASL (FASLG) | 2.30 | 0.0054 |
| LY9 | 2.12 | 0.0041 |
| LYN | 2.08 | 0.0031 |
| TNFRSF21 | 2.07 | 0.0028 |
| CD27 | 1.98 | 0.021 |
| CD244 | 1.92 | 0.007 |
| ARG1 | 1.90 | 0.021 |
| CCL17 | 1.82 | 0.0037 |
| CD40L (CD40LG) | 1.79 | 0.00079 |
| FGFBP1 | 1.75 | 0.043 |
| CD48 | 1.74 | 0.0065 |
| ANG1 (ANGPT1) | 1.59 | 0.0066 |
| CCL23 | 1.49 | 0.017 |
| PDCD1 | 1.19 | 0.018 |
| DCN | 0.27 | 0.0047 |
| ERBB4 | 0.26 | 0.024 |
| IGF1R | 0.18 | 0.0093 |
| **^#^** part of boxed dendogram branches |  | *** p-value** after Bonferroni corr. |

**Supplementary Table 6.** Proteins in the PEA-panels used (for more information: www.olink.com)

| **Protein name** | **Gene name** | **UniProt Accession** | **UniProt/ SwissProt ID** | **PEA panel Oncology II** | **PEA panel Immune_ Oncology I** | **Excluded proteins from  model (>25% NAN)** |
| --- | --- | --- | --- | --- | --- | --- |
| Tyrosine-protein kinase ABL1 | ABL1 | P00519 | ABL1_HUMAN | X |  |  |
| Adenosine deaminase | ADA | P00813 | ADA_HUMAN |  | X |  |
| Disintegrin and metalloproteinase domain-containing protein 8 (ADAM 8) | ADAM8 | P78325 | ADAM8_HUMAN | X |  |  |
| A disintegrin and metalloproteinase with thrombospondin motifs 15 (ADAM-TS 15) | ADAMTS15 | Q8TE58 | ATS15_HUMAN | X |  |  |
| Adhesion G-protein coupled receptor G1 | ADGRG1 | Q9Y653 | AGRG1_HUMAN |  | X | X |
| Angiopoietin-1 (ANG-1) | ANGPT1 | Q15389 | ANGP1_HUMAN |  | X |  |
| Angiopoietin-2 (ANG-2) | ANGPT2 | O15123 | ANGP2_HUMAN |  | X | X |
| Annexin A1 | ANXA1 | P04083 | ANXA1_HUMAN | X |  |  |
| Amphiregulin (AR) | AREG | P15514 | AREG_HUMAN | X |  |  |
| Arginase-1 | ARG1 | P05089 | ARGI1_HUMAN |  | X |  |
| Carbonic anhydrase 9 | CA9 | Q16790 | CAH9_HUMAN | X | X | X |
| Caspase-8 (CASP-8) | CASP8 | Q14790 | CASP8_HUMAN |  | X |  |
| C-C motif chemokine 13 | CCL13 | Q99616 | CCL13_HUMAN |  | X |  |
| C-C motif chemokine 17 | CCL17 | Q92583 | CCL17_HUMAN |  | X |  |
| C-C motif chemokine 19 | CCL19 | Q99731 | CCL19_HUMAN |  | X |  |
| C-C motif chemokine 2 | CCL2 | P13500 | CCL2_HUMAN |  | X |  |
| C-C motif chemokine 20 | CCL20 | P78556 | CCL20_HUMAN |  | X |  |
| C-C motif chemokine 23 | CCL23 | P55773 | CCL23_HUMAN |  | X |  |
| C-C motif chemokine 3 | CCL3 | P10147 | CCL3_HUMAN |  | X |  |
| C-C motif chemokine 4 | CCL4 | P13236 | CCL4_HUMAN |  | X |  |
| C-C motif chemokine 7 | CCL7 | P80098 | CCL7_HUMAN |  | X | X |
| C-C motif chemokine 8 | CCL8 | P80075 | CCL8_HUMAN |  | X |  |
| CD160 antigen | CD160 | O95971 | BY55_HUMAN | X |  |  |
| C-type lectin domain family 4 member K | CD207 | Q9UJ71 | CLC4K_HUMAN | X |  | X |
| Natural killer cell receptor 2B4 | CD244 | Q9BZW8 | CD244_HUMAN |  | X |  |
| CD27 antigen | CD27 | P26842 | CD27_HUMAN | X | X |  |
| Programmed cell death 1 ligand 1 (PD-L1) | CD274 | Q9NZQ7 | PD1L1_HUMAN |  | X | X |
| T-cell-specific surface glycoprotein CD28 | CD28 | P10747 | CD28_HUMAN |  | X |  |
| T-cell surface glycoprotein CD4 | CD4 | P01730 | CD4_HUMAN |  | X |  |
| Tumor necrosis factor receptor superfamily member 5 | CD40 | P25942 | TNR5_HUMAN |  | X |  |
| CD40 ligand (CD40-L) | CD40LG | P29965 | CD40L_HUMAN |  | X |  |
| CD48 antigen | CD48 | P09326 | CD48_HUMAN | X |  |  |
| T-cell surface glycoprotein CD5 | CD5 | P06127 | CD5_HUMAN |  | X |  |
| CD70 antigen | CD70 | P32970 | CD70_HUMAN | X | X | X |
| CD83 antigen (hCD83) | CD83 | Q01151 | CD83_HUMAN |  | X |  |
| T-cell surface glycoprotein CD8 alpha chain | CD8A | P01732 | CD8A_HUMAN |  | X |  |
| Cyclin-dependent kinase inhibitor 1 | CDKN1A | P38936 | CDN1A_HUMAN | X |  |  |
| Carcinoembryonic antigen-related cell adhesion molecule 1 | CEACAM1 | P13688 | CEAM1_HUMAN | X |  |  |
| Carcinoembryonic antigen-related cell adhesion molecule 5 | CEACAM5 | P06731 | CEAM5_HUMAN | X |  | X |
| Carboxypeptidase E (CPE) | CPE | P16870 | CBPE_HUMAN | X |  |  |
| **Protein name (cont. Tab 5)** | **Gene name** | **UniProt Accession** | **UniProt/ SwissProt ID** | **PEA panel Oncology II** | **PEA panel Immune_ Oncology I** | **Excluded proteins from  model (>25% NAN)** |
| Cornulin | CRNN | Q9UBG3 | CRNN_HUMAN | X |  |  |
| Cytotoxic and regulatory T-cell molecule | CRTAM | O95727 | CRTAM_HUMAN |  | X | X |
| Macrophage colony-stimulating factor 1 (CSF-1) | CSF1 | P09603 | CSF1_HUMAN |  | X |  |
| Cathepsin L2 | CTSV | O60911 | CATL2_HUMAN | X |  |  |
| Fractalkine | CX3CL1 | P78423 | X3CL1_HUMAN |  | X |  |
| Growth-regulated alpha protein | CXCL1 | P09341 | GROA_HUMAN |  | X |  |
| C-X-C motif chemokine 10 | CXCL10 | P02778 | CXL10_HUMAN |  | X |  |
| C-X-C motif chemokine 11 | CXCL11 | O14625 | CXL11_HUMAN |  | X |  |
| Stromal cell-derived factor 1 (SDF-1) | CXCL12 | P48061 | SDF1_HUMAN |  | X | X |
| C-X-C motif chemokine 13 | CXCL13 | O43927 | CXL13_HUMAN | X | X |  |
| VEGF coregulated chemokine 1 | CXCL17 | Q6UXB2 | VCC1_HUMAN | X |  |  |
| C-X-C motif chemokine 5 | CXCL5 | P42830 | CXCL5_HUMAN |  | X |  |
| Interleukin-8 (IL-8) | CXCL8 | P10145 | IL8_HUMAN |  | X |  |
| C-X-C motif chemokine 9 | CXCL9 | Q07325 | CXCL9_HUMAN |  | X |  |
| Protein CYR61 | CYR61 | O00622 | CYR61_HUMAN | X |  |  |
| Decorin | DCN | P07585 | PGS2_HUMAN |  | X |  |
| Delta-like protein 1 | DLL1 | O00548 | DLL1_HUMAN | X |  |  |
| Interleukin-27 subunit beta (IL-27 subunit beta) | EBI3 | Q14213 | IL27B_HUMAN |  | X |  |
| Pro-epidermal growth factor (EGF) | EGF | P01133 | EGF_HUMAN | X | X |  |
| Ephrin type-A receptor 2 | EPHA2 | P29317 | EPHA2_HUMAN | X |  |  |
| Receptor tyrosine-protein kinase erbB-2 | ERBB2 | P04626 | ERBB2_HUMAN | X |  |  |
| Receptor tyrosine-protein kinase erbB-3 | ERBB3 | P21860 | ERBB3_HUMAN | X |  |  |
| Receptor tyrosine-protein kinase erbB-4 | ERBB4 | Q15303 | ERBB4_HUMAN | X |  |  |
| Endothelial cell-specific molecule 1 (ESM-1) | ESM1 | Q9NQ30 | ESM1_HUMAN | X |  |  |
| FAS-associated death domain protein | FADD | Q13158 | FADD_HUMAN | X |  |  |
| Tumor necrosis factor ligand superfamily member 6 | FASLG/FasL | P48023 | TNFL6_HUMAN | X | X |  |
| Fc receptor-like B | FCRLB | Q6BAA4 | FCRLB_HUMAN | X |  | X |
| Fibroblast growth factor 2 (FGF-2) | FGF2 | P09038 | FGF2_HUMAN |  | X |  |
| Fibroblast growth factor-binding protein 1 (FGF-BP) | FGFBP1 | Q14512 | FGFP1_HUMAN | X |  |  |
| Vascular endothelial growth factor receptor 3 (VEGFR-3) | FLT4 | P35916 | VGFR3_HUMAN | X |  | X |
| Folate receptor alpha (FR-alpha) | FOLR1 | P15328 | FOLR1_HUMAN | X |  |  |
| Folate receptor gamma (FR-gamma) | FOLR3 | P41439 | FOLR3_HUMAN | X |  |  |
| Furin | FURIN | P09958 | FURIN_HUMAN | X |  |  |
| Glypican-1 | GPC1 | P35052 | GPC1_HUMAN | X |  | X |
| Transmembrane glycoprotein NMB | GPNMB | Q14956 | GPNMB_HUMAN | X |  |  |
| Granzyme A | GZMA | P12544 | GRAA_HUMAN |  | X |  |
| Granzyme B | GZMB | P10144 | GRAB_HUMAN | X | X |  |
| Granzyme H | GZMH | P20718 | GRAH_HUMAN | X | X |  |
| Hepatocyte growth factor | HGF | P14210 | HGF_HUMAN | X | X |  |
| Heme oxygenase 1 (HO-1) | HMOX1 | P09601 | HMOX1_HUMAN |  | X |  |
| ICOS ligand | ICOSLG | O75144 | ICOSL_HUMAN | X | X | X |
| **Protein name (cont. Tab 5)** | **Gene name** | **UniProt Accession** | **UniProt/ SwissProt ID** | **PEA panel Oncology II** | **PEA panel Immune_ Oncology I** | **Excluded proteins from  model (>25% NAN)** |
| Interferon beta (IFN-beta) | IFNB1 | P01574 | IFNB_HUMAN |  | X | X |
| Interferon gamma (IFN-gamma) | IFNG | P01579 | IFNG_HUMAN |  | X | X |
| Interferon gamma receptor 1 (IFN-gamma receptor 1) | IFNGR1 | P15260 | INGR1_HUMAN | X |  |  |
| Insulin-like growth factor 1 receptor | IGF1R | P08069 | IGF1R_HUMAN | X |  |  |
| Interleukin-10 (IL-10) | IL10 | P22301 | IL10_HUMAN |  | X | X |
| Interleukin-12 subunit alpha (IL-12A) | IL12A | P29459 | IL12A_HUMAN |  | X | X |
| Interleukin-12 subunit alpha (IL-12A) | IL12A | P29459 | IL12A_HUMAN |  | X | X |
| Interleukin-12 subunit beta (IL-12B) | IL12B | P29460 | IL12B_HUMAN |  | X |  |
| Interleukin-12 receptor subunit beta-1 (IL-12 receptor subunit beta-1) | IL12RB1 | P42701 | I12R1_HUMAN |  | X | X |
| Interleukin-13 (IL-13) | IL13 | P35225 | IL13_HUMAN |  | X | X |
| Interleukin-18 (IL-18) | IL18 | Q14116 | IL18_HUMAN |  | X |  |
| Interleukin-1 alpha (IL-1 alpha) | IL1A | P01583 | IL1A_HUMAN |  | X |  |
| Interleukin-2 (IL-2) | IL2 | P60568 | IL2_HUMAN |  | X | X |
| Interleukin-21 (IL-21) | IL21 | Q9HBE4 | IL21_HUMAN |  | X | X |
| Interleukin-33 (IL-33) | IL33 | O95760 | IL33_HUMAN |  | X |  |
| Interleukin-4 (IL-4) | IL4 | P05112 | IL4_HUMAN |  | X | X |
| Interleukin-5 (IL-5) | IL5 | P05113 | IL5_HUMAN |  | X | X |
| Interleukin-6 (IL-6) | IL6 | P05231 | IL6_HUMAN | X | X |  |
| Interleukin-7 (IL-7) | IL7 | P13232 | IL7_HUMAN |  | X | X |
| Integrin alpha-V | ITGAV | P06756 | ITAV_HUMAN | X |  |  |
| Integrin beta-5 | ITGB5 | P18084 | ITB5_HUMAN | X |  |  |
| Vascular endothelial growth factor receptor 2 (VEGFR-2) | KDR | P35968 | VGFR2_HUMAN | X | X |  |
| Kit ligand | KITLG | P21583 | SCF_HUMAN | X |  |  |
| Kallikrein-11 (hK11) | KLK11 | Q9UBX7 | KLK11_HUMAN | X |  |  |
| Kallikrein-13 | KLK13 | Q9UKR3 | KLK13_HUMAN | X |  |  |
| Kallikrein-14 (hK14) | KLK14 | Q9P0G3 | KLK14_HUMAN | X |  |  |
| Kallikrein-8 (hK8) | KLK8 | O60259 | KLK8_HUMAN | X |  |  |
| Natural killer cells antigen CD94 | KLRD1 | Q13241 | KLRD1_HUMAN |  | X | X |
| Lysosome-associated membrane glycoprotein 3 (LAMP-3) | LAMP3 | Q9UQV4 | LAMP3_HUMAN |  | X | X |
| Galectin-1 (Gal-1) | LGALS1 | P09382 | LEG1_HUMAN | X | X |  |
| Galectin-9 (Gal-9) | LGALS9 | O00182 | LEG9_HUMAN |  | X |  |
| T-lymphocyte surface antigen Ly-9 | LY9 | Q9HBG7 | LY9_HUMAN | X |  |  |
| Tyrosine-protein kinase Lyn | LYN | P07948 | LYN_HUMAN | X |  |  |
| Ly6/PLAUR domain-containing protein 3 | LYPD3 | O95274 | LYPD3_HUMAN | X |  |  |
| Midkine (MK) | MDK | P21741 | MK_HUMAN | X |  |  |
| Methionine aminopeptidase 2 (MAP 2) | METAP2 | P50579 | MAP2_HUMAN | X |  |  |
| Melanoma-derived growth regulatory protein | MIA | Q16674 | MIA_HUMAN | X |  |  |
| MHC class I polypeptide-related sequence A (MIC-A) | MICA | Q29983 | MICA_HUMAN | X | X | X |
| MHC class I polypeptide-related sequence B (MIC-B) | MICB | Q29980 | MICB_HUMAN | X | X |  |
| Macrophage metalloelastase (MME) | MMP12 | P39900 | MMP12_HUMAN |  | X |  |
| **Protein name (cont. Tab 5)** | **Gene name** | **UniProt Accession** | **UniProt/ SwissProt ID** | **PEA panel Oncology II** | **PEA panel Immune_ Oncology I** | **Excluded proteins from  model (>25% NAN)** |
| Matrilysin | MMP7 | P09237 | MMP7_HUMAN |  | X |  |
| Mesothelin | MSLN | Q13421 | MSLN_HUMAN | X |  | X |
| Mucin-16 (MUC-16) | MUC16 | Q8WXI7 | MUC16_HUMAN | X |  |  |
| Natural cytotoxicity triggering receptor 1 | NCR1 | O76036 | NCTR1_HUMAN |  | X | X |
| Nectin-4 | NECTIN4 | Q96NY8 | NECT4_HUMAN | X |  |  |
| Nitric oxide synthase, endothelial | NOS3 | P29474 | NOS3_HUMAN |  | X |  |
| 5'-nucleotidase (5'-NT) | NT5E | P21589 | 5NTD_HUMAN | X |  |  |
| Programmed cell death protein 1 (Protein PD-1) | PDCD1 | Q15116 | PDCD1_HUMAN |  | X | X |
| Programmed cell death 1 ligand 2 (PD-1 ligand 2) | PDCD1LG2 | Q9BQ51 | PD1L2_HUMAN |  | X | X |
| Platelet-derived growth factor subunit B (PDGF subunit B) | PDGFB | P01127 | PDGFB_HUMAN |  | X |  |
| Placenta growth factor (PlGF) | PGF | P49763 | PLGF_HUMAN |  | X |  |
| Podocalyxin | PODXL | O00592 | PODXL_HUMAN | X |  |  |
| Pancreatic prohormone | PPY | P01298 | PAHO_HUMAN | X |  | X |
| Pleiotrophin (PTN) | PTN | P21246 | PTN_HUMAN |  | X | X |
| Proto-oncogene tyrosine-protein kinase receptor Ret | RET | P07949 | RET_HUMAN | X |  |  |
| R-spondin-3 | RSPO3 | Q9BXY4 | RSPO3_HUMAN | X |  | X |
| Protein S100-A11 | S100A11 | P31949 | S10AB_HUMAN | X |  |  |
| Protein S100-A4 | S100A4 | P26447 | S10A4_HUMAN | X |  |  |
| Secretory carrier-associated membrane protein 3 (Secretory carrier membrane protein 3) | SCAMP3 | O14828 | SCAM3_HUMAN | X |  |  |
| Syndecan-1 (SYND1) | SDC1 | P18827 | SDC1_HUMAN | X |  |  |
| Seizure 6-like protein | SEZ6L | Q9BYH1 | SE6L1_HUMAN | X |  |  |
| Mothers against decapentaplegic homolog 5 (MAD homolog 5) | SMAD5 | Q99717 | SMAD5_HUMAN | X |  |  |
| SPARC | SPARC | P09486 | SPRC_HUMAN | X |  |  |
| T-cell leukemia/lymphoma protein 1A | TCL1A | P56279 | TCL1A_HUMAN | X |  |  |
| Angiopoietin-1 receptor | TEK | Q02763 | TIE2_HUMAN |  | X | X |
| Tissue factor pathway inhibitor 2 (TFPI-2) | TFPI2 | P48307 | TFPI2_HUMAN | X |  |  |
| Protransforming growth factor alpha | TGFA | P01135 | TGFA_HUMAN | X |  |  |
| Transforming growth factor beta-1 (TGF-beta-1) | TGFB1 | P01137 | TGFB1_HUMAN |  | X | X |
| TGF-beta receptor type-2 (TGFR-2) | TGFBR2 | P37173 | TGFR2_HUMAN | X |  |  |
| Toll-like receptor 3 | TLR3 | O15455 | TLR3_HUMAN | X |  |  |
| Tumor necrosis factor | TNF | P01375 | TNFA_HUMAN |  | X | X |
| Tumor necrosis factor receptor superfamily member 12A | TNFRSF12A | Q9NP84 | TNR12_HUMAN |  | X | X |
| Tumor necrosis factor receptor superfamily member 19 | TNFRSF19 | Q9NS68 | TNR19_HUMAN | X |  |  |
| Tumor necrosis factor receptor superfamily member 21 | TNFRSF21 | O75509 | TNR21_HUMAN |  | X |  |
| Tumor necrosis factor receptor superfamily member 4 | TNFRSF4 | P43489 | TNR4_HUMAN | X | X |  |
| Tumor necrosis factor receptor superfamily member 6B | TNFRSF6B | O95407 | TNF6B_HUMAN | X |  |  |
|  |  |  |  |  |  |  |
| **Protein name (cont. Tab 5)** | **Gene name** | **UniProt Accession** | **UniProt/ SwissProt ID** | **PEA panel Oncology II** | **PEA panel Immune_ Oncology I** | **Excluded proteins from  model (>25% NAN)** |
| Tumor necrosis factor receptor superfamily member 9 | TNFRSF9 | Q07011 | TNR9_HUMAN |  | X |  |
| Tumor necrosis factor ligand superfamily member 10 | TNFSF10 | P50591 | TNF10_HUMAN | X | X |  |
| Tumor necrosis factor ligand superfamily member 12 | TNFSF12 | O43508 | TNF12_HUMAN |  | X |  |
| Tumor necrosis factor ligand superfamily member 13 | TNFSF13 | O75888 | TNF13_HUMAN | X |  |  |
| Tumor necrosis factor ligand superfamily member 14 | TNFSF14 | O43557 | TNF14_HUMAN |  | X |  |
| TNF-related apoptosis-inducing ligand | TRAIL | P50591 | TNF10_HUMAN | X | X |  |
| Alpha-taxilin | TXLNA | P40222 | TXLNA_HUMAN | X |  |  |
| Vascular endothelial growth factor A (VEGF-A) | VEGFA | P15692 | VEGFA_HUMAN | X | X |  |
| Vascular endothelial growth factor C (VEGF-C) | VEGFC | P49767 | VEGFC_HUMAN |  | X | X |
| WAP four-disulfide core domain protein 2 | WFDC2 | Q14508 | WFDC2_HUMAN | X |  |  |
| Wnt inhibitory factor 1 (WIF-1) | WIF1 | Q9Y5W5 | WIF1_HUMAN | X |  | X |
| Vimentin | VIM | P08670 | VIME_HUMAN | X |  |  |
| WNT1-inducible-signaling pathway protein 1 (WISP-1) | WISP1 | O95388 | WISP1_HUMAN | X |  |  |
| Xaa-Pro aminopeptidase 2 | XPNPEP2 | O43895 | XPP2_HUMAN | X |  | X |
